# The pathogenic mechanism of *Mycobacterium tuberculosis*: implication for new drug development

**DOI:** 10.1186/s43556-022-00106-y

**Published:** 2022-12-22

**Authors:** Weizhu Yan, Yanhui Zheng, Chao Dou, Guixiang Zhang, Toufic Arnaout, Wei Cheng

**Affiliations:** 1 Division of Respiratory and Critical Care Medicine, Respiratory Infection and Intervention Laboratory of Frontiers Science Center for Disease-Related Molecular Network, State Key Laboratory of Biotherapy, West China Hospital of Sichuan University, Chengdu, 610041 China; 2 Division of Gastrointestinal Surgery, Department of General Surgery and Gastric Cancer center, West China Hospital, Sichuan University, No. 37. Guo Xue Xiang, Chengdu, 610041 China; 3 Kappa Crystals Ltd., Dublin, Ireland; 4 MSD Dunboyne BioNX, Co. Meath, Ireland

**Keywords:** *Mycobacterium tuberculosis*, Resistance, Persistence, Structure-based drug design, Pathogenesis

## Abstract

*Mycobacterium tuberculosis* (*Mtb*), the causative agent of tuberculosis (TB), is a tenacious pathogen that has latently infected one third of the world’s population. However, conventional TB treatment regimens are no longer sufficient to tackle the growing threat of drug resistance, stimulating the development of innovative anti-tuberculosis agents, with special emphasis on new protein targets. The *Mtb* genome encodes ~4000 predicted proteins, among which many enzymes participate in various cellular metabolisms. For example, more than 200 proteins are involved in fatty acid biosynthesis, which assists in the construction of the cell envelope, and is closely related to the pathogenesis and resistance of mycobacteria. Here we review several essential enzymes responsible for fatty acid and nucleotide biosynthesis, cellular metabolism of lipids or amino acids, energy utilization, and metal uptake. These include InhA, MmpL3, MmaA4, PcaA, CmaA1, CmaA2, isocitrate lyases (ICLs), pantothenate synthase (PS), Lysine-ε amino transferase (LAT), LeuD, IdeR, KatG, Rv1098c, and PyrG. In addition, we summarize the role of the transcriptional regulator PhoP which may regulate the expression of more than 110 genes, and the essential biosynthesis enzyme glutamine synthetase (GlnA1). All these enzymes are either validated drug targets or promising target candidates, with drugs targeting ICLs and LAT expected to solve the problem of persistent TB infection. To better understand how anti-tuberculosis drugs act on these proteins, their structures and the structure-based drug/inhibitor designs are discussed. Overall, this investigation should provide guidance and support for current and future pharmaceutical development efforts against mycobacterial pathogenesis.

## Introduction

Tuberculosis (TB) is the second leading cause of mortality among infectious diseases worldwide in the twenty-first century, with two million deaths annually [1, 2]. Moreover, it is estimated that one-third of the world’s population harbor a latent TB infection by the *Mycobacterium tuberculosis* (*Mtb*) complex (including *Mycobacterium tuberculosis*, *Mycobacterium africanum, Mycobacterium bovis*, *Mycobacterium caprae*, *Mycobacterium microti*, *Mycobacterium pinnipedii,* and *Mycobacterium canettii*) [3]. The incidence of TB has been increasing due to several factors, including the HIV epidemic [4], the widespread emergence of drug-resistant *Mtb* strains [multidrug-resistant *Mtb* (MDR-*Mtb*), extensively drug-resistant *Mtb* (XDR-*Mtb*) and totally drug-resistant *Mtb* (TDR-*Mtb*)] [5–7], as well as the lack of medical/drug compliance. Unfortunately, existing TB treatment regimens have not been updated to keep up with these challenges, hence are insufficient to tackle these drug-resistant forms. Therefore, there is an urgent need to develop new anti-tuberculosis drugs that are active against drug-resistant bacteria but, more importantly, kill persistent bacteria.

**Fig. 1:**
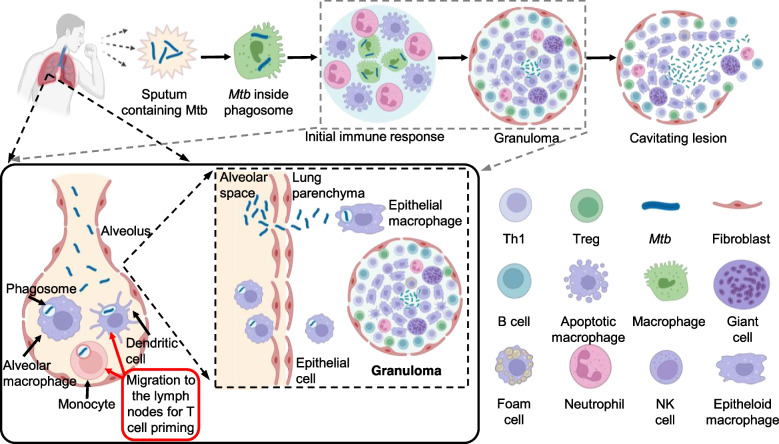
Pathogenesis of *Mycobacterium tuberculosis*. Infection begins when *Mtb* from aerosols or sputum undergoes phagocytosis in the lung reaching tissue-resident alveolar macrophages and dendritic cells. *Mtb* then undergoes a transient period of unrestricted intracellular replication, during which infected cells migrate to local draining lymph nodes. Once there, *Mtb* can infect other areas of the lungs by infecting other host cells. With the onset of cellular immunity, a local proinflammatory response leads to the recruitment of additional monocytes and lymphocytes, which in turn cluster around the infected macrophages, forming what is known as a granuloma. Granuloma is a pathological hallmark of tuberculosis, and it is thought that *Mtb* persists in a prolonged state of delayed or arrested replication at this site. If *Mtb* multiplies too much, the granuloma will not be able to control the infection and *Mtb* will eventually spread to other organs, including the brain. At this stage, *Mtb* can enter the bloodstream or re- enter the respiratory tract to be released causing new infections. The elements in the figure were drawn using biorender online tool (https://biorender.com)

Using special virulence factors and/or essential genes to develop vaccines, drugs and diagnostic reagents against TB is a worthwhile approach. The *Mtb* H37Rv genome consists of 4.4 × 10^6^ bp (65.6% GC), encoding ~4000 predicted proteins [8]. These annotated proteins are involved in multiple cellular metabolic pathways, including DNA or RNA methylation, RNA processing, protein processing, lipid synthesis, membrane assembly, cell division, and cytoplasmic and membrane transfer steps of peptidoglycan synthesis, in which numerous metabolic pathways are closely related to the pathogenicity of *Mtb*. A unique feature of the *Mtb* genome is that over 200 proteins (6% of the total) participate in the metabolism of fatty acids, among which approximately 100 are predicted to function in the β-oxidation of fatty acids. This large number of *Mtb* enzymes may be related to the ability of this pathogen to grow in specific tissues of the infected host, in which fatty acids act as the major carbon source [9]. Thus, considerable drugs or inhibitors targeting the biosynthesis of mycolic acids are reported and used in clinical research [10]. For instance, anti-tuberculosis drugs isoniazid and ethionamide have been proven to inhibit the biosynthesis of mycolic acids (Table 1) and exert their function by inactivating the reductase activity of the enoyl-acyl-carrier protein (InhA). In addition, the *Mtb* genome encodes five separate type VII secretion systems (TSSS). Among these five, the secretion system Esx-1 is well characterized, and this system could promote the necrotic death of infected cells and the recruitment of macrophages, allowing the intracellular *Mtb* to be released to the extracellular space and uptaken by the freshly recruited adjacent phagocytes, ultimately resulting in intracellular bacterial population expansion [27–30] (Fig. 1). The critical role of the secretion system Esx-1 has been applied into the attenuated vaccine strain *Mycobacterium bovis* BCG [31–33].

**Table 1:** An overall summary of available anti-tuberculosis drugs and their characteristics

Drug name	Targets	Mechanisms of action	Genes involved in resistance
Rifampicin	RNA polymerase, β subunit	Inhibits RNA synthesis [11, 12]	*rpoB*
Isoniazid	Enoyl- [acyl-carrier protein] reductase (InhA)	Inhibits mycolic acid biosynthesis and affects the metabolism of DNA, lipid, carbohydrate, and NAD	*katG, inhA, ahpC, ndh*
Pyrazinamide [13]	S1 component of the 30S ribosomal subunit	Inhibits translation and trans-translation, acidifies cytoplasm	*pncA, FAS-I*
Ethambutol	Arabinosyl transferase	Arabinogalactan biosynthesis inhibition	*embCAB*
Kanamycin [14]	30S ribosomal subunit	Inhibition of protein synthesis	*rrs*
Amikacin [15]	30S ribosomal subunit	Inhibition of protein synthesis	*rrs*
Capreomycin [15]	Interbridge B2a	Inhibition of protein synthesis	*rrs, tlyA*
Streptomycin	S12 and 16S rRNA components of 30S ribosomal subunit	Inhibition of protein synthesis	*rpsL,rrs*
Fluoroquinolones [16]	DNA gyrase and DNA topoisomerase	Inhibition of DNA supercoiling	*gyrA, gyrB, IfrA*
Ethionamide	Enoyl- [acyl-carrier protein] reductase (InhA)	Inhibition of mycolic acid biosynthesis	*inhA, etaA/ethA*
Cycloserine [17]	D-alanine racemase and ligase	Inhibits peptidoglycan biosynthesis	*alrA, ddl*
Para-amino salicylic acid [18]	Thymidylate synthase (ThyA) and dihydropteroate synthase	Inhibits folic acid and iron metabolism	*thyA, folC*
Clofazimine [19]	Exact target not yet known	Release of reactive oxygen species (ROS) and cell member disruption	Rv0678, and *mmpL5*
Linezolid [20]	50S ribosomal subunit	Inhibits protein synthesis	*rplC*
β-lactam/β-lactamase inhibitors	β-lactamases	Cell wall disruption via peptidoglycan modulation	*blaC*, Rv0194
Thiacetazone	Flavin monooxygenase EtaA	Inhibition cyclopropanation of cell wall mycolic acids	*etaA*
Clarithromycin	50S ribosomal subunit	Protein synthesis inhibition	/
Bedaquiline [21]	ATP synthase	Inhibition of mitochondrial ATP synthase	Rv0678, *atpE*
Delamanid [22]	Exact target unknown	Inhibits mycolic acid synthesis and cell respiration	Rv3547
Pretomanid^a^ [23]	Exact target unknown	Inhibits cell wall synthesis and causes respiratory poisoning	/
Delpazolid^a^	50S ribosomal subunit	Protein synthesis inhibition	/
Sutezolid^a^	50S ribosomal subunit	Protein synthesis inhibition	/
SQ109^a^ [24]	MmpL3	Inhibits cell wall synthesis	/
PBTZ169^a^ [25]	DprE1	Inhibits cell wall synthesis	/
Q203^a^ [26]	Cytochrome *bc1* complex	Inhibits ATP synthesis	/

^a^these drugs are in clinical trials

In this review, we focus on some enzymes that are essential for the pathogenicity of TB to summarize their structures and structure-based inhibitor/drug design. Several enzymes [(e.g., enoyl-acyl carrier protein reductase (InhA), mycolic acid transports enzyme (MmpL3), mycolic acid methyltransferase (MmaA4), and mycolic acid cyclopropane synthases (PcaA, CmaA1, CmaA2)] that participate in the mycolic acid pathway [34–37], are highlighted in this work. Enzymes involved in amino acid metabolism [lysine-ε amino transferase (LAT), Isopropylmalate isomerase (LeuD)], lipid metabolism [isocitrate lyases (ICLs), pantothenate synthetase (PS)], metal uptake (IdeR), energy metabolism [catalase-peroxidase (KatG), fumarate hydratase (Rv1098c)], pyrimidine biosynthetic [cytidine triphosphate synthetase (PyrG)], and transcription regulation (PhoP), as well as the cell secretion [glutamine synthetase (GlnA1)] are also summarized. These enzymes are either therapeutic drug targets or potential drug targets.

## Tuberculosis therapeutics

Currently, the standard TB treatment regimen for drug-susceptible TB consists of a 6–9-month course of first-line anti-tuberculosis drugs (isoniazid, rifampicin, ethambutol, and pyrazinamide). However, long-term therapies are not only significantly toxic, but also frequently lead to poor compliance of patients, and in turn, facilitate the development of drug-resistant TB. These conventional anti-tuberculosis drugs are insufficient to completely eradicate bacteria that remain in a state of latent infection. For example, standard TB therapy is ineffective in controlling MDR-TB (resistant to at least two first-line drugs). Treatment of XDR-TB (characterized as MDR-TB with additional resistance to any fluoroquinolone and at least one of the three second-line prescribed drugs) requires the use of third-line anti-TB drugs, which are less effective or have higher side effects [38, 39]. TDR-TB infection, the most severe form of infection, is caused by *Mtb* strains that are resistant to all of the first- and second-line drugs. To address the issue of therapeutic failure, constant attention has been focused on this area. The world health organization (WHO) has designated Group 5 antibiotics, including repurposed drugs and drugs with unclear efficacy or an unclear role in the treatment of DR-TB, such as thiacetazone, high-dose isoniazid, clofazimine, linezolid, amoxicillin plus clavulanate, macrolides, carbapenem, and thioridazine [40]. In addition to chemotherapy, immunotherapeutic approaches (e.g., DNA vaccines, and cytokines) combined with chemotherapy are also providing options for the improved treatment of TB [41–43]. The currently available anti-tuberculosis drugs, the targets, the mode of action, and the genes associated with the drug resistance are listed in Table 1, including some drug candidates with high anti-tuberculosis potential at clinical trials.

## Validated and potential targets of anti-tuberculosis drugs

We focus on enzymes that participate in the cellular metabolism of *Mtb*, including mycolic acid and nucleotide biosynthesis, and metabolism of lipids, amino acids, energy utilization, and metal uptake. Other enzymes, such as the transcriptional regulator PhoP, and Glutamine synthetase are also included.

### Enzymes involved in cellular metabolism

#### Enzymes associated with mycolic acid biosynthesis

Mycolic acids (MAs), α-branched β-hydroxylated long chain fatty acids (C_70_-C_90_), are major constituents of the mycobacterial cell envelope [44, 45]. They may be covalently bound to cell wall arabinogalactan, rendering the *Mtb* cell envelope extremely hydrophobic and impermeable to a variety of compounds [46–51], and thus function as a physical barrier against the host immune system and exogenous antibiotics [52]. In addition, the metabolism of MAs is also highly associated with the physiology, virulence, and pathogenicity of Mycobacterium [36, 37, 53, 54]. Accordingly, targeting the enzymes involved in the metabolism of MAs is an excellent strategy for the development of effective anti-tuberculosis agents. At present, several effective anti-tuberculosis drugs, such as isoniazid [55], ethionamide, thiacetazone, and delamanid, have been shown to inhibit the biosynthesis of MAs.

Fatty acids cannot be scavenged from the host and must be synthesized de novo [56]. The biosynthesis of MAs begins with the synthesis of saturated C_16–18, 22–26_ fatty acids by the multifunctional fatty acid synthase I (FAS-I), which is then extended to C_48–62_ by the FAS-II multienzyme system. At the same time, it is modified by a group of eight S-adenosylmethionine-dependent methyltransferases (SAM-MTs) in two distinct positions (distal and proximal positions on the meromycolic chain) [57, 58]. The *cis* double bonds, which are necessary for the process of decorating, may be converted into cyclopropane by MmaA2 and PcaA [36, 59], or converted into a *trans* double bond by UmaA1 [60], or hydrated into hydroxylated mycolates by MmaA4 [53, 57, 61, 62]. The product of MmaA4 can be further modified into keto- and methoxy-MAs by MmaA3 or an unidentified dehydrogenase [53], respectively. The decorated MAs are finally translocated into the periplasm by MmpL3 [63]. Several essential enzymes involved in the biosynthesis of MAs (InhA, MmaA4, MmpL3, PacA, CmaA1, and CmaA2) that have been identified or may become potential targets of anti-tuberculosis drugs are highlighted.

##### Enoyl-acyl carrier protein reductase (InhA)

**Fig. 2:**
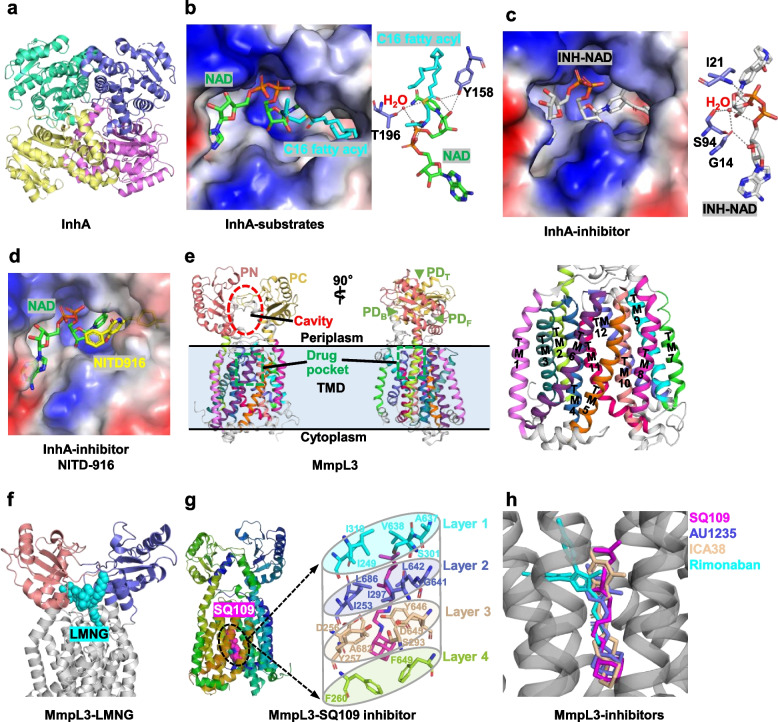
Enzymes associated with mycolic acid biosynthesis. **a** Overall structure of Enoyl-acyl carrier protein reductase (InhA, PDB ID: 4TRM). **b** Structure of the C16 fatty acyl and NAD+ substrates bound to InhA protein (PDB ID: 1BVR). Hydrogen bonds between the active site of InhA and the bound C16 fatty acyl substrate are shown as gray dashes drawn between interacting atoms. **c** Structure of InhA protein with the INH-NAD adduct (PDB ID: 2IDZ). The oxygen O9 of the phosphate of the INH-NAD adduct forms a hydrogen bond with the main-chain nitrogen atom of Ile21 and a hydrogen bond with a well-ordered water molecule. The water molecule is part of a hydrogen-bonding network formed by interactions between the side-chain oxygen atom of Ser94, the main-chain oxygen of Gly14 and the oxygen atoms O3 and O9 of the INH-NAD adduct. The same water molecule is within hydrogen-bonding distance of the main-chain nitrogen atoms of Ile21. **d** Electrostatic surface of InhA in a complex with inhibitor NITD-916. **e** Cartoon representation of the MmpL3 crystal structure. The subdomain PN and PC intertwine to create a central cavity, which connects to three openings, a funnel at the top (PDT), an opening in the front (PDF) and an opening at the back (PDB) of the headpiece. TMD, transmembrane domain. **f** Structure of MmpL3 in a complex with lauryl maltose neopentyl glycol (LMNG). **g** MmpL3 inhibitor binding pocket for SQ109. The four layers of residues surround SQ109 like a cylinder. **h** Superposition of SQ109-bound, AU1235- bound, ICA38-bound, and Rimonabant-bound structures. It shows that all the inhibitors have similar binding positions. The electrostatic potential in all figures was computed using the APBS tools in PyMol (http://www.pymol.org/)

As a crucial biosynthetic enzyme involved in MAs, InhA catalyzes the NADH-dependent reduction of long-chain trans-2-enoyl-ACP in type II fatty acids of *Mtb* [64, 65]. More importantly, the *Mtb* InhA has no human ortholog [66], and as such, there might be less risk of inhibitor toxicity occurrence. Therefore, InhA has been developed into a well-validated target for the treatment of *Mtb*, especially for the frontline or second line antitubercular drugs isoniazid and ethionamide [67]. The apo-InhA structure is a tetrameric form with a characteristic of short-chain dehydrogenase/reductase (SDR) (Fig. 2a) [68, 69]. Each protomer contains a canonical fold of enoyl-ACP reductase, wherein several α-helices and β-strands of the central Rossmann fold form a deep crevice [70]. The complex structure of InhA with NAD^+^ and a C_16_ fatty acyl substrate demonstrates how each substrate recognizes InhA (Fig. 2b). The NAD^+^ is perpendicular to the β-strands of the Rossmann fold. A fatty acyl substrate adopts a general “U-shaped” conformation and is embedded in a deep substrate-binding crevice composed of several hydrophobic residues (Ala198, Met199, Ala201, Ile202, Leu207, Ile215, and Leu218) [71]. The hydrogen bond between the thioester carbonyl oxygen of a fatty acyl substrate and the side chain hydroxyl oxygen of Tyr158 is the only direct hydrogen between the acyl substrate and InhA [71]. Furthermore, several hydrogen bonds between the fatty acyl substrate and NAD^+^/ a water molecule, also contribute to the stability of the complex.

The first-line tuberculosis drug isoniazid (INH) has been applied in clinical treatment since the 1950s and has been validated to target InhA [72]. It is a prodrug, which requires the *Mtb* catalase-peroxidase KatG activation to generate its acyl radical or acyl anion form [67], which subsequently reacts with the cellular NAD^+^, resulting in an INH-NAD adduct and consequently interferes with the biosynthesis of mycolic acids. This inhibition mechanism is also found in other anti-tuberculosis drugs such as ethionamide and propionamide [55, 73]. In the ternary complex structure of InhA-NAD-INH (Fig. 2c), a covalent interaction between the carbonyl carbon of the acyl group of INH and the carbon at position four of the nicotinamide ring of NAD occurs [72]. The acyl group of INH forms π-π stacking interactions with the side chain of Phe149. Several hydrogen bonds (between the phosphate of the NAD and the Ile21 of InhA and a water molecule) together with a hydrogen-bonding network (consisting of Ser94, Gly14 of InhA, O3 and O9 of NAD) maintain the stability of the INH-NAD adduct [72]. Therefore, the S94A mutation in InhA not only reduces the affinity for NADH, but also produces resistance to INH by disruption of the hydrogen-bonding network [72].

Multiple INH-resistant clinical strains have been found to contain a KatG-associated mutation [74], hence exploring direct inhibitors of InhA that do not require bioactivation such as isoniazid or ethionamide, is receiving increased attention. Various effective inhibitors of InhA have been screened, identified, and optimized through a structure-based approach, such as pyrazole derivatives, indole-5-amides [75], alkyl diphenyl ethers, triazole-based diphenyl ethers [76, 77], triclosan derivatives [78], diazaborines [79], acrylamides [80], 4-hydroxy-2-pyridones [65], prothionamide [73], methyl-thiazole series [52], and pyrrolidine carboxamides [81]. The binding sites of these direct InhA inhibitors can be divided into several regions: the catalytic or active site, the hydrophobic pocket that accommodates the substrate’s long alkyl chains, and the solvent-exposed site that is termed the size-limited region [52, 82, 83]. In addition, these inhibitors can be subdivided into cofactor-independent, and cofactor-dependent. The inhibition mechanism of some representative inhibitors is described here. A representative NADH-dependent inhibitor that occupies the enoyl-substrate binding site is NITD-916 which belongs to the 4-hydroxy-2-pyridone family [65]. In the ternary complexes of InhA-NADH-NITD-916 (Fig. 2d), a variety of interactions stabilize the complex structure, including π-stacking (between the pyridine ring of NITD-916 and NADH), hydrogen bonding (between the 4-hydroxy group of NITD-916 and the 2′-hydroxyl moiety of the nicotinamide ribose sugar, and Tyr158 of InhA), and hydrophobic interactions (between the dimethyl cyclohexyl group of NITD-916 and the fatty acyl substrate). Distinguished from NITD-916 which binds to the InhA-NADH product complex, most direct InhA inhibitors bind to the InhA-NAD product complex, including triclosan [75], alkyl diphenyl ethers [76], and pyrrolidine carboxamides [81]. Some cofactor-dependent inhibitors such as pyridomycin can simultaneously occupy the NADH and lipid substrate-binding pocket of InhA [84, 85]. In addition, some inhibitors function in a cofactor-independent manner, such as AN12855 which also occupies both the NADH and substrate binding sites [79].

##### Mycobacterial membrane protein larger transporters (MmpL)

MmpL transporter families are responsible for transporting fatty acids and other lipids from the production site to the cell wall, which is necessary for mycobacterial replication and viability [86, 87]. Mycobacterial genomes encode 13 MmpL proteins, all of which are necessary for the virulence of mycobacteria [88–93]. For example, MmpL5 and MmpL7 can effectively eliminate anti-tubercular drugs, including anti-MDR-TB drug bedaquiline [94, 95]. As a trehalose monomycolate (TMM) flippase, MmpL3 translocates intracellular MAs (in the form of TMM) from the cytoplasm to periplasm [93, 96], which is a process driven by the proton-motive force (PMF) [97]. In the periplasmic space, a mycolate chain from one TMM molecule is transferred to another molecule to form trehalose dimycolate (TDM; cord factor), or covalently linked to arabinogalactan-peptidoglycan layer to produce mycolyl arabinogalactan peptidoglycan (mAGP) [98, 99]. MmpL3 is essential for shuttling of TMM across the cell membrane [90, 93], and the inactivation of MmpL3 by small-molecule inhibitors or genetic methods was shown to be bactericidal [63, 100, 101]. Thus, MmpL3 is an excellent target for the discovery of anti-tubercular drugs [102–107].

*Mtb* MmpL3 is structurally distinct from all known Resistance-Nodulation-Division (RND) protein superfamily members, which is ubiquitous among bacteria, archaea, and eukaryotes [8, 90, 108, 109]. *Mtb mmpl3*, encoding for a protein with 61%  sequence identity with that encoded by *Mycobacterium smegmatis* (*Msmg*) *mmpl3*, can rescue the viability of the *Msmg mmpl3* null mutant [93]. In addition, many significant insights into the interactions between *Mtb* MmpL3 and its inhibitors are also reported using the *Msmg* ortholog [103, 110, 111]. Recently, a C-terminal truncated *Mtb* MmpL3 (residues 1 to 753; MmpL3_1–753_) has been determined by cryo-electron microscopy (Cryo-EM) (Fig. 2e) [112]. In other studies, the proline-rich C-terminal domain (residues 733 to 1013) of *Mtb* MmpL3 was prone to proteolysis and was not necessary for molecular function [111, 113]. The transmembrane domain (TMD) of MmpL3_1–753_ contains 12 transmembrane helices (TMs 1–12) organized as two sequence-contiguous bundles (TMs 1–6 and 7–12). Two periplasmic flexible loops (loop 1 and 2) are connected to TMs 1–2 in the N-terminal half of MmpL3_1–753 _(residues 37–166), and TMs 7–8 in the C-terminal half of the molecule (residues 415–544), respectively, generating two periplasmic subdomains PN and PC. Both PN and PC subdomains display an α-β-α-β-α-β topology, with the first α helix of each contributing to the tertiary structure of the adjacent loop. A singular periplasmic domain (PD) is observed in the interface of PN and PC, which acts as the pseudo-symmetry axis of the molecule. Like the structure of *Msmg* MmpL3, *Mtb* MmpL3 also has a large cavity enclosed by the PD, which is presumably related to the translocation of TMM [113]. This periplasmic central cavity has three distinct apertures orientating to the periplasm (PD_F_, PD_B,_ and PD_T_), which are gated by a combination of charged and hydrophilic residues. In the structure of MmpL3_1–753_, the detergent lauryl maltose neopentyl glycol (LMNG) was immobilized within this central cavity in a splayed conformation, where the central vestibule sequesters the alkyl chains away from the periplasm. The proximate hydrophilic openings (PD_F,_ PD_B_, and PD_T_) bind to the polar head group of LMNG (Fig. 2f). MmpL3 protein can recognize various lipids, including TMM (but not TDM), phosphatidylethanolamine (PE), phosphatidylglycerol (PG), phosphatidylinositol (PI), and cardiolipin (CDL) [111]. All of these adopt a segmentation-binding mode like that of LMNG, which permits specific molecules to enter or exit. Superimposition of the complex structures of MmpL3 with different lipid substrates revealed that their conformations have different periplasmic central cavity volumes, which is induced by the rigid movement of subdomain PN, and the corresponding rearrangement of several TMs [114].

A series of *Msmg* MmpL3 inhibitors with diverse chemical scaffolds have been reported [115–122] and can be divided into nine classes, including ethylenediamines [24, 115, 123], urea derivatives [93, 124], indolcarboxamides [117, 121, 125], pyrroles and pyrazoles [126], tetrahydropyrazolopyrimidine carboxamides [127, 128], spirocycles [127, 128], piperidinol derivatives [129, 130], benzimidazoles [131], and HC2091 [132]. Some of these compounds were observed to synergize with existing anti-tubercular drugs [118, 121, 133]. Multiple research advancements of the complex structure of MmpL3 and its inhibitors indicate that most MmpL3 inhibitors are in the central pocket within the TMD and exert their activity by disrupting hydrogen bonding interactions between two conserved Asp-Tyr pairs, resulting in blocking the proton motive force that drives substrate translocation. An example is SQ109 which clearly illustrates the mechanism of action of these inhibitors. SQ109, an ethylenediamine compound, shows strong bactericidal activity against all forms of *Mtb*, including drug-resistant clinical strains [115, 134]. It is a promising preclinical anti-tuberculosis drug candidate and has been studied in phase 2b/3 clinical trials [24]. The crystal structure of MmpL3 in the complex with SQ109 shows that SQ109 is bound to the center of the TMs bundle in an extended conformation (Fig. 2g) [103]. Upon inhibitor binding, most of the six C-terminal TMs (TMs 7–12) were induced to move away from the center of the TM region, generating a pocket with a volume of 282 Å^3^ to accommodate SQ109. The interactions between SQ109 and MmpL3 are mainly hydrophobic, and the interface between SQ109 and MmpL3 can be divided into four layers (layer 1–4). The geranyl tail is inserted into the upper hydrophobic region of the pocket and stabilized by the hydrophobic constituents of layer 1 (Ile249, Ile319, Ala637, Val638, and Ser301) and layer 2 (Ile253, Ile297, Gly641, Leu642, and Leu686). Meanwhile, the side chain of Leu642 moves 3.1 Å away from the center of the TM bundle, which provides space for the methyl group protruding from the SQ109 backbone. In layer 3, two amide nitrogen groups of SQ109 interact with the side chain of Asp645 by hydrogen bonds. The adjacent Ser293 participates in the hydrogen bonding network, stabilizing Tyr257 and Asp645. However, due to the movement of TMs, the hydrogen bond (between Asp256 and Tyr646) observed in the apo structure disappeared in the complex structure of MmpL3 with SQ109. Consequently, both Asp-Tyr pairs (Asp256-Tyr646 and Asp645-Tyr257), which are known to be involved in the proton-relay network, are broken due to the binding of SQ109. The adamantine group of SQ109 resides in the hydrophobic bottom pocket (layer 4), which is surrounded by hydrophobic residues Phe260 and Phe649. The phenyl groups of Phe260 and Phe649 undergo significant conformational changes upon SQ109 binding, and their phenyl ring rotates by 7 Å, forming a V-shaped structure, which matches with the adamantine group of SQ109.

Aside from SQ109, several inhibitors of MmpL3, including AU1235, ICA38, Rimonabant, NITD-349, and SPIRO, have shown a strong bactericidal ability against *Mtb*, and have been proven to target MmpL3 [117, 125, 128, 135]. Structures of MmpL3 bound with these inhibitors show that these inhibitors bind in the same pocket as that of SQ109 (Fig. 2h) [103]. Structurally, all these inhibitors have a hydrophobic head and tail, and central nitrogen atoms, which form hydrogen bonds with the conserved Asp645, destroying the proton relay and thereby blocking the proton motive force used for substrate translocation. However, due to the diversity of the inhibitors’ skeletons, the specific interactions of these compounds are quite different. For example, the bulky tri-fluorophenyl group of AU1235 that occupies the hydrophobic subsite on the top of the pocket can generate more hydrophobic interactions than does the geranyl tail of SQ109; and the distance moved by C-terminal TMs bundle induced by inhibitors, as well as the volume of the corresponding binding pocket are also different among these inhibitors. Notably, a recent study on the mutational landscape of drug resistance of *Mtb* variants shows that most mutation sites are either concentrated or near to (< 10 Å) the drug-binding pocket [112]. Therefore, it is suggested that alternative inhibitors targeting other domains should be developed [112].

##### Mycolic acid methyltransferase (MmaA4)

**Fig. 3:**
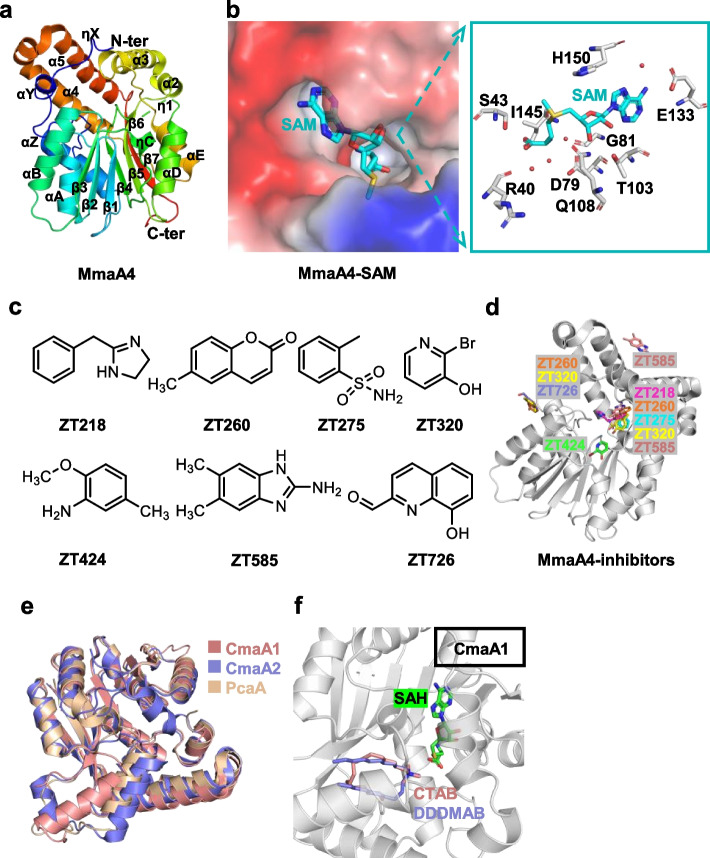
Enzymes participating in the specific modification of mycolic acid. **a** Structure of MmaA4 (PDB ID: 2FK7). **b** Structure of MmaA4-SAM (PDB ID: 2FK8). The electrostatic potential was computed using the APBS tools in PyMol (http://www.pymol.org/). **c**, **d** Structure of MmaA4-inhibitors (PDB ID: 7Q2B-H). **e** Superposition of CmaA1, CmaA2 and PcaA (PDB ID: 1KP9, 1KPI, 1L1E). **f** Structure of CmaA1-SAH-CTAB/DDDMAB (PDB ID: 1KPG, 1KPH)

Compared with other SAM-MTs, the structures of apo-MmaA4 and MmaA4-SAM complex both contain a typical core SAM-MT fold [a central seven-stranded β-sheet (β3-β2-β1-β4-β5-β7-β6) with three helices flanking on each side] and several individual components [including an α helical at the N-terminal, and a set of four antiparallel α helical (α2-α5) between strands β6 and β7] (Fig. 3a) [136, 137]. A hydrophobic tunnel of MmaA4 (residues 180 to 216, called α2-α3 motif) protrudes from the protein surface to the cofactor binding site and covers α2 and α3 helices, and the sequence connecting α3-αE displaying the same basic/hydrophobic patches as other SAM-MTs [136, 138]. This α2-α3 motif has closely related biochemical functions to SAM-MTs, such as determining whether the decoration reaction occurs at the proximal or distal position, accommodating hydrophobic substrates, and is compatible with its meromycolate substrate processed protein [acyl carrier protein from *Mtb* (AcpM)] with an acidic/hydrophobic patch [138, 139]. The pore size of the tunnel is determined by the steric obstruction generated by three hydrophobic residues, Ile201, Val205, and Leu214 [136]. The SAM cofactor locates in a crevice at the top of the central β-sheet and is stabilized by the polar/van der Waals interaction (Fig. 3b) [136]. SAM-binding induces the structural rearrangement of the segment (residues 147 to 154) from the disordered loop to the short η1-helix (residues 148 to 150) [136]. In addition, the superimposed structures of apo-MmaA4 and MmaA4-SAM binary complex also demonstrated that some unique structural elements only exist in the latter, such as the helices ηx at the N-terminus. The above structural information can be applied to guide the design of competitive inhibitors of SAM cofactors and analogues.

By screening a library of fragments, several bound ligands (ZT218, ZT260, ZT275, ZT320, ZT424, ZT585, ZT726) of MmaA4 have been identified (Fig. 3c). These ligands have two binding regions (one located in a deep crevice that accommodates substrate/ SAM cofactor, and the other located on the surface of protein), and two different binding modes (Fig. 3d) [58]. Fragment ZT218, ZT260, ZT585, and ZT424 have the same binding mode as the SAM cofactor [58, 136]. However, two fragments ZT275 and ZT320, which are located at the substrate-binding site of MmaA4, induce rearrangement of a segment (residues 147–154 loop) to generate a new conformation, and cause the inability of the cofactor to be compatible with the MmaA4, which indicates that the allosteric inhibitors of MmaA4 can be designed [58]. In the complex structures of MmaA4 with ZT275/ZT320, the residue Phe148 of helix η1 is pushed away from its original positions (about 10 Å) in the complex structure of MmaA4-SAM, and the position of adenine moiety of the cofactor is occupied by residues Glu149 and His150 [58]. A similar conformational change of the helix η1 is also observed in the complex structure of MmaA4-ZT424, and this compound bound to the position of the adenine moiety of SAM cofactor by van der Waals interactions [58]. Apart from the position of the substrate-binding, fragments ZT260, ZT320, and ZT585 can also bind to the different regions of MmaA4 surface [58]. Among these three compounds, the planar aromatic ring of ZT260 and ZT320 is intercalated between the guanidinium group of the two arginine residues, and forms a perpendicular aromatic-aromatic interaction with the indole moiety of Tyr84; while ZT585 is located between two protein molecules through van der Waals/hydrogen bonds [58]. Generally, based on the structural insights into the above-mentioned fragments with MmaA4, chimeric inhibitors with improved binding affinities shall be designed.

##### Mycolic acid cyclopropane synthases

Based on the specific modification at the distal and proximal positions of the acyl chain, *Mtb* MAs can be divided into three classes, including *α*-, keto-, and methoxymycolates [138]. The *α*-mycolates contain a *cis* cyclopropane ring at both positions, while keto- and methoxymycolates have oxygenated functional groups at the distal position and a *cis* or *trans* cyclopropane ring at the proximal position [138]. The hydroxylation modification catalyzed by MmaA4 has been discussed above, and here the cyclopropanation modification is discussed. The cyclopropanation of MAs has been proven to be closely related to the pathogenicity, persistence, anti-oxidative stress, fluidity, and permeability of mycobacterial cell wall [34, 53, 140]. For example, the cyclopropanation catalyzed by PcaA (also named UmaA2) is essential for the nucleation morphology of *Mtb* [138]. Outside of PcaA, cyclopropane synthases CmaA1 and CmaA2, are also responsible for the cyclopropanation of MAs, among which CmaA1 catalyzes cyclopropanation at the distal position, while CmaA2 catalyzes the modification at the proximal position, which is similar to PcaA [35, 138, 141, 142]. All three proteins are SAM-dependent methyltransferases, which catalyze methyl transfer through the general acid and base mechanism.

These three cyclopropane synthases share 50–75% sequence identity with several other homologous MA methyltransferases (including MmaA1–4, UmaA1 [8, 138]), one of which CmaA1 is the search model for structural analysis of MmaA4 protein determined by molecular replacement [136]. Superimposition of the structures of CmaA2, PcaA, and CmaA1 illustrates that there is little difference in their overall fold, and all contain a core seven-stranded antiparallel β sheet (β3-β2-β1-β4-β5-β7-β6) with α helices flanking either side (Fig. 3e). Given its characteristics and common feature, CmaA1 is a representative to describe how these cyclopropane synthases recognize their cofactor and lipid substrates. The ternary structures of CmaA1-SAH-CTAB and CmaA1-SAH-DDDMAB share the same overall fold as that of apo-CmaA1, excluding residues 137 to 144, and the first 20 residues at the N-terminal end [138]. When the ternary complex is formed (Fig. 3f), the fragment (residues 137 to 144) undergoes a conformational change from a flexible loop to a 3_10_ helix, and this helix forms a narrow channel, making the cofactor and lipid substrate binding sites connected [138]. The changed conformation pushes the β5-α11 loop (residues 170 to 210) away from the cofactor binding site (5–10 Å), which leads to the lipid-binding pocket being closer to the surface and makes it shallower [138]. The cationic substrates with an alkyl chain are filled in the hydrophobic/basic tunnel in a U-shaped conformation. Only hydrophobic interactions occur between the protein and the lipid substrate. However, multiple sets of hydrogen bonds and van der Waals interactions stabilize the cofactor on the top of the central β-sheet. Residues involved in the interactions between protein and cofactor or lipid substrates are conserved among these three mycolic acid cyclopropane synthases. The α9 helix, the only distinct region among the three cyclopropane synthases, is involved in the formation of the entry point of the lipid-binding pocket and may be related to the position of cyclopropanation modification in MAs acyl chain [138]. This speculation is based on the fact that the α9 helix forms a planar surface in the proximal enzymes (CmaA2 and PcaA), but forms a domed, protruded surface in the distal enzyme (CmaA1). The planar surface is more conducive to the binding of acyl carrier protein and subsequent catalytic reaction.

#### Enzymes involved in Lipid metabolism

##### Isocitrate lyases (ICLs)

Given that conventional anti-mycobacterial drugs have little effect on the persistent bacteria, it is urgent to identify novel targets that are highly associated with persistent infection, to develop new antimycobacterial agents. During the chronic stages of *Mtb* infection, lipids (especially odd-chain fatty acids and cholesterol) are preferentially utilized as the primary carbon source [143–146], simultaneously triggering a corresponding metabolic shift from tricarboxylic acid (TCA) cycle to glyoxylate shunt and methylcitrate cycle [147, 148]. Glyoxylate shunt and methylcitrate cycle exist in most prokaryotes, lower eukaryotes, and plants, but not in vertebrates [149]. The essential magnesium-dependent isocitrate lyase (two isoforms, ICL1 and ICL2) is a key enzyme for two pathways [147, 150–152]. These two isocitrate lyases reversibly catalyze the retro-aldol cleavage of isocitrate and methylcitrate to form glyoxylate and pyruvate, respectively, as well as the same product succinate. Then, acetyl-CoA is added to the metabolite glyoxylate to form malate through malate synthase (encoded by the gene *glcB* [153]). ICLs are essential for *Mtb* survival [147, 150], because the activity of ICLs increases dramatically as the cells reach the stationary phase [154], and when *Mtb* infects human inflammatory macrophages, its mRNA level also increases [155–157]; disruption of *icl* leads to the growth impairment of *Mtb* [157]. Additionally, it was validated that ICLs are associated with bacterial virulence [151], and antibiotic tolerance [158]. Taken together, the essentiality of ICLs and the absence of homologous enzymes in humans make them attractive therapeutic targets against latent infections [159–161].

**Fig. 4:**
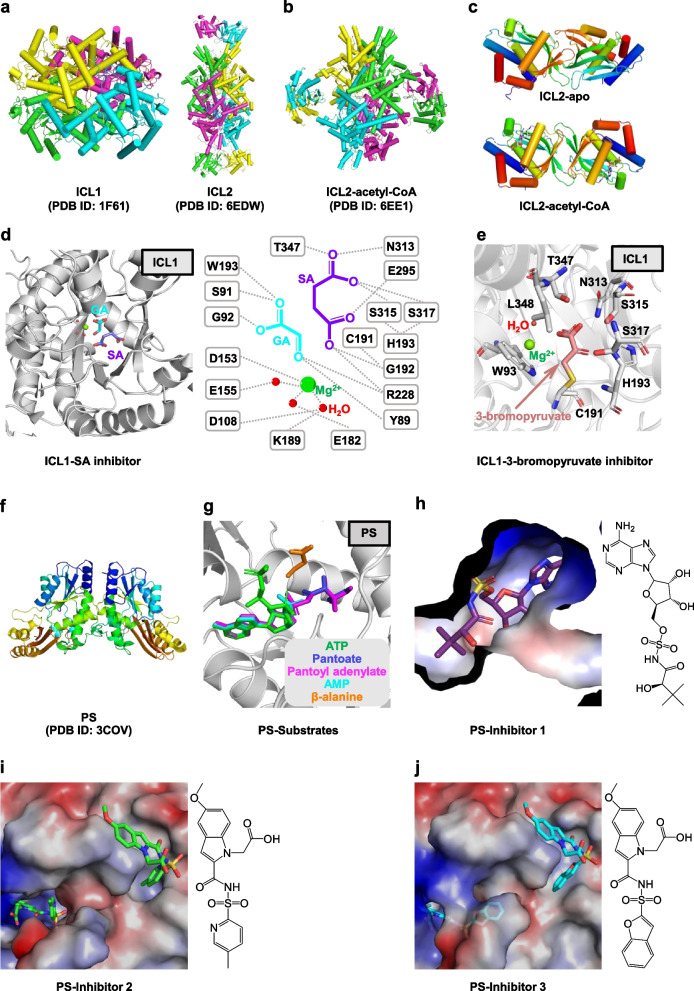
Enzymes associated with cellular lipid metabolism. **a** Structures of ICL1 homotetramer (Left, PDB ID: 1F61) and ICL2 (Right, PDB ID: 6EDW). ICL2 forms an elongated structure with C-terminal dimers at each end of the structure. **b** Striking structural rearrangement of ICL2 upon binding to acetyl- CoA (PDB ID: 6EE1). In both **a** and **b**, each monomer is shown in different color. **c** The dimeric association of the C-terminal domains in the ligand-free (top) and acetyl-CoA-bound (bottom) ICL2. Acetyl-CoA is shown as sticks. **d** Left: structure of the ternary complex of the ICL1 with glyoxylate (GA) and 3-nitropropionate (shown as succinate, SA) (PDB ID: 1F8I). Right: Schematic diagram of ICL interactions with glyoxylate and succinate. **e** Structure of ICL1 in a complex with 3-bromopyruvate (PDB ID: 1F8M). Pyruvyl moeity (purple) is formed by covalently binding 3-bromopyruvate to C191 of ICL1. **f** Structure of pantothenate synthetase (PS, PDB ID: 3COV). A side view of the dimer structure shows that it resembles the shape of a butterfly. **g** Structures of PS in a complex with different substrates (ATP, pantoate, pantoyl adenylate, AMP and β-alanine), respectively. **h**-**j** Structure of PS in a complex with different inhibitors. Inhibitor 1, 5′-O-{[(2R)-2-hydroxy-3,3-dimethylbutanoyl]sulfamoyl}adenosine; Inhibitor 2, (5-methoxy-2-{[(5-methylpyridin-2-yl)sulfonyl]carbamoyl}-1H-indol-1-yl)acetic acid; Inhibitor 3, 2-(2- (benzofuran-2-Ylsulfonylcarbamoyl)-5-methoxy-1H-indol-1-yl)acetic acid. The electrostatic potential in all figures was computed using the APBS tools in PyMol (http://www.pymol.org/)

Though ICL1 and ICL2 share 27% sequence identity, their overall structures are quite different [162, 163]. ICL1 is a homo-tetramer (Fig. 4a), and each subunit consists of 14 α-helices and 14 β-strands. The core of the structure consists of eight α helices (α4-α11) and eight β-strands (β2-β5, β8, β12-β14), forming an α/β-barrel [(βα)_2_α(βα)_5_β] [162]. Two adjacent subunits are connected to each other by the exchange of C-terminal regions containing helices α12 and α13. ICL1 possesses an active site loop (residues 185 to 196), which contains a conserved catalytic motif K189KCGH193. More structural details are described below in the complex structures of ICL1 with various inhibitors. Unlike ICL1, there are a few studies related to the structure of ICL2. ICL2 packs as a homo-tetramer with an elongated conformation (Fig. 4a) [163]. Each protomer is made up of two distinct domains, a catalytic N-terminal domain (residues 1 to 592), and a regulatory C-terminal domain (residues 607 to 766), connected by a flexible linker (residue 591 to 602). The N-terminal domain consists of an α/β-barrel central structure (similar to ICL1) and a unique helical substructure (α10-α16; residues 278–427). The C-terminal domains from two subunits associate with each other at each end of the ICL2 structure, assembling into a barrel-like structure. Additionally, ICL2 also has an active site loop containing residues Lys213 to His217. The activity of ICL2 is activated by the binding of acetyl-CoA or propionyl-CoA [163], along with a remarkable structure rearrangement in the binding process [163]. In the complex structure of ICL2/ acetyl-CoA (Fig. 4b-c), the C-terminal domain from one monomer moves 77 Å towards the center of ICL2, and rotates about 176°, forming a new dimer with the C-terminal domain from the opposite monomer [163]. This allosteric activation induced by acetyl-CoA or propionyl-CoA is a crucial mechanism during persistent infection with lipids as the primary carbon source.

A series of ICL1 inhibitors have been reported, ranging from small molecules to peptides [164–171], such as 3-bromopyruvate [170], 3-nitropropionate (shown as succinate, SA) [171], and 2-vinyl-D-isocitrate (2-VIC) [167]. Most of these can be classified as covalent inhibitors, with an advantage that they do not easily promote bacterial drug resistance [172–174]. Some inhibitors, including 2-VIC, are pro-drugs, and require a base-catalyzed retro-aldol cleavage by ICL1 to form an intermediate. Generally, these inhibitors adopt the same inhibition mode that covalently modifies the active site residue Cys191 to form a covalent adduct, thus trapping the enzyme in a closed configuration, and the solvent cannot access the active site [162]. Here, 3-bromopyruvate and 3-nitropropionate are taken as examples to describe their inhibition mechanism, these two inhibitors are good compounds for the structure-based drug design. In the ternary complex structure of ICL1 (C191S)/glyoxylate (GA)/SA (Fig. 4d), GA is coordinated by the active site Mg^2+^ and several hydrogen bonds with residues Ser91, Gly92, Trp193, and Arg228. One carboxylate of 3-nitropropionate (3-nitropropionate was replaced by succinate in the Fourier density map) makes specific hydrogen bonds with residues Asn313, Glu295, Arg228, and Gly192, while the second carboxylate forms hydrogen bonds with Thr347, Asn313, Ser315, and Ser317 [171]. The C2 and C3 methylene carbons of 3-nitropropionate are stacked with residues Trp93, Thr347, and Leu348. In the complex structure of ICL1/3-bromopyruvate (Fig. 4e), the pyruvyl moiety makes hydrogen bonds with residues His193, Asn313, Ser315, Ser317, Thr347, and a water molecule, as well as a covalent linkage with Cys191, resulting in an ICL1-inhibitor covalent adduct. In this complex structure, solvent molecules occupy the glyoxylate-binding site. Inhibitors induced conformational changes. In the apo structure of ICL1, the active site loop (residues 185–196) preferentially maintains an open and solvent-accessible conformation [162], where Cys191 is positioned at a considerable distance away from other catalytic residues. Upon binding the inhibitor, significant conformational changes take place in two regions. Firstly, the active-site loop moves 10–15 Å and adopts a closed, solvent-inaccessible conformation [162], thus preventing the substrate from approaching the catalytic site. However, there is enough space for the inhibitor to migrate and react with Cys191 in the closed active site. Secondly, the last 18 residues (residues 411 to 428) at the C-terminus of the adjacent subunit move and lie on the top of the active site loop, further locking it into the closed conformation.

Some other special properties of remaining covalent inhibitors are summarized below. 2-VIC was cleaved by ICL1 to produce an enzyme-bound Michael acceptor, 2-vinylglyoxylate (2-VG), which subsequently combines with Cys191 to form a reversible, covalent adduct [167]. Additionally, 2-VIC has a time-dependent inactivation effect on ICL2. Itaconate, structurally analogous to succinate, covalently inhibits two ICL isoforms by catalyzing the conjugate addition at the cysteine residue (Cys191 of ICL1 and Cys215 of ICL2) [175]. To overcome some defects of these inhibitors, such as low cell permeability, toxicity, and easy elimination or reversal of inhibition in the presence of free thiols (e.g. DTT and glutathione), more durable covalent inhibitors of ICLs are explored. Examples include (2*R*,3*S*)-2-hydroxy-3-(nitromethyl)-succinic acid (5-NIC) and *cis*-2,3-Epoxy-succinic acid (*cis*-Eps). 5-NIC undergoes retro-aldol cleavage to form glyoxylate and 3-nitropropionic acid (3-NP) [161], and the latter reacts with the Cys191 of ICL1 to form a more stable and irreversible ICL1-thiohydroxamate adduct [161]. *cis*-Eps, the most potent irreversible covalent inhibitor of ICL1 yet discovered, can bind to the succinate subsite of ICL1 and form a covalent adduct with the proximity of Cys191 [176].

##### Pantothenate synthetase (PS)

Pantothenate (vitamin B5) is a necessary precursor for the biosynthesis of coenzyme A (CoA) and acyl carrier proteins (ACP). These two proteins play crucial roles in numerous cellular processes, such as energy and fatty acid metabolism [177, 178]. Microorganisms and plants are capable of de novo pantothenate synthesis, while mammals can only obtain this fundamental nutrient through their routine diet [179]. Consequently, the pantothenate biosynthetic pathway provides potential targets for antimicrobial agents [9, 180]. The pantothenate biosynthetic pathway consists of four steps, catalyzed by the product of *panB*, *panC, panD*, and *panE* genes respectively [181, 182]. The *panBCDE* cluster encodes ketopantoate hydroxymethyltransferase, pantothenate synthetase (PS), aspartate-1-decarboxylase, and ketopantoate reductase, respectively. PS catalyzes the final step of pantothenate biosynthesis, a magnesium-ATP-dependent condensation of pantoate with β-alanine to generate pantothenate. There are two consecutive reactions: from ATP and pantoate to form an enzyme-bound intermediate (pantoyl adenylate), and then the intermediate is nucleophilically attacked by β-alanine to produce pantothenate and AMP [183, 184]. Pantothenate biosynthesis is necessary for the virulence of *Mtb*, and it was found that *Mtb* pantothenate auxotrophy with *panC* (Rv3602c) and *panD* (Rv3601c) gene defects was highly attenuated in mice infection models [185]. And an attenuated *Mtb* strain, with both *panCD* and the primary attenuating mutations of the Bacilli Calmette-Guérin (BCG) strain removed, was investigated as a potential human vaccine candidate to prevent TB [186]. Therefore, there is a growing interest in using *Mtb* PS as an antitubercular target, and a series of methods have been used to find the inhibitors of this enzyme [187, 188].

The dimer structure of *Mtb* PS is butterfly-shaped, which is similar to the structure of *E. coil* PS enzyme (Fig. 4f) [177, 189]. Each subunit is composed of two domains: a large N-terminal domain (residues 1 to 186) employing a Rossmann fold, and a smaller two-layered C-terminal domain (residues 187 to 290) with a helical layer on top of a three-stranded antiparallel β-sheet. The enzymatic active-site cavity is located in a cleft between strands β2 and β6 and is partially covered by β-strands from the C-terminal domain. This closed conformation contrasts with the open form of the *E. coil* PS, whose C-terminal domain is typically away from the active-site cavity [189]. A flexible region (residues 74 to 88) forms a wall to the active site cavity (termed flexible wall), while it becomes ordered upon binding of the reaction intermediate bound, thus serving as a gate to the active-site cavity. Additionally, four arginine residues (Arg198, Arg132, Arg273, Arg278) form a positively charged region covering the active-site cavity, which might be used to manipulate the negatively charged substrates [177].

Aside from the residues on the flexible wall, no significant conformational changes are observed between the structures of apo *Mtb* PS and its various complexes. Notably, different crystal packing environments lead to different substrate occupancy at two active sites [177, 190]. The complex structures of PS with five ligands (ATP, pantoate, pantoyl adenylate, AMP, β-alanine) are referred to describe their interactions with the enzyme (Fig. 4g). (1) Substrate ATP is tightly bound to the bottom of the active-site cavity through hydrophobic and hydrogen bonding interactions. Its adenine group is flanked by Gly46 and Lys160, where the N1 and N6 atoms make hydrogen bonds with the main-chain atoms of Val187 and Met195/Val187, respectively. The N3 atom faces the hydrophobic side chains of Val184 and Leu50. The hydroxyl groups of ribose make hydrogen bonds with the side chain of Asp161 and several main chain atoms from the bottom of the active-site cavity (including Gly158, Phe156, and Pro38). The phosphate groups turn back towards the top of the active-site cavity and are located near the N-terminal end of helices α2 and 3_10_7. Its α-phosphate forms water-mediated hydrogen bonds with Met40, Gly41, and His47; β- and γ-phosphate groups form salt bridges with Lys160 and Arg198, respectively. The bridging oxygen between the α- and β- phosphates makes a hydrogen bond with the Met40. The cofactor magnesium ion binds to the ATP and has a perfect octahedral coordination with three ligands from oxygen atoms of the phosphate groups and the other three from water molecules. (2) Another substrate, pantoate, occupies the hydrophobic pocket within the active-site cavity, and its carboxyl oxygen is close to the α-phosphorus atom of ATP, allowing for in-line nucleophilic attack. The pantoate molecule is tightly bound through hydrogen bonds (with the side chains of Gln72 and Gln164) and hydrophobic interactions (with side chains of Pro38, Phe157, and Met40). (3) The reaction intermediate pantoyl adenylate is tightly bound to the bottom of the active-site cavity in an almost linear conformation, suggesting that non-reactive analogs of pantoyl adenylate are effective inhibitors of the PS enzyme. The binding mode of pantoyl adenylate is equal to that of the pantoate and the adenosine group of ATP. Its α-phosphate group forms a covalent bond with the carboxyl group of pantoate moiety, and a hydrogen bond with the amide nitrogen of Met40, simultaneously. (4) The binding modes of product AMP are similar to those of ATP. However, the phosphate group of AMP has torsional flexibility and is slightly rotated with respect to the α-phosphate of ATP. (5) The phosphate group of pantoyl adenylate probably acts as an anchor for the initial binding of β-alanine by providing hydrogen-bonding and/or favorable charge-charge interactions [177, 190]. The upper part of the active-site cavity is occupied by β-alanine, but its binding affinity is weaker than that of the other molecules. Its amino group makes water-mediated hydrogen bonds with the phosphate group of the intermediate, and its carboxyl group makes a hydrogen bond with the side chain of Gln72, fixed by the Asn69 through a hydrogen bond. In addition, its carboxyl group forms charge-charge interactions and π-electron interactions with the side chains of Arg198 and His135.

Currently, the research on PS inhibition mainly focused on the synthesis of non-reactive analogues of the reaction intermediate [191, 192], or the identification of hits by high-throughput screening coupled with structure-based validation [193–198]. The inhibition mechanism of several inhibitors is discussed here. (1) 5′-O-[(2R)-2-hydroxy-3,3-dimethylbutanoyl]-sulfamoyl-adenosine (inhibitor 1), an analogue of pantoyl adenylate, exhibits dissociation and inhibition constants in a nanomolar scale [191]. The binding mode of this inhibitor is nearly identical to that of pantoyl adenylate (Fig. 4h) [191, 199], and its adenine and ribose moiety make the same interactions as that of pantoyl adenylate. In addition, its sulphonamide group interacts with the side chain of His44 and the backbone amide of Met40. An ordered network of water molecules, which is found around the sulphonamide group, mediates hydrogen bonds between the carboxylate moiety of Asp161 and the sulphonamide and carbonyl group of inhibitor 1. The terminal hydroxyl group forms hydrogen bonds with the side chains of Gln72 and Gln164, and its replacement with an amine would significantly weaken the binding affinity. (2) (5-methoxy-2-[(5-methylpyridin-2-yl) sulfonyl] carbamoyl-1H-indol-1-yl) acetic acid (inhibitor 2) derived from the fragment-growing of compound 5-methoxyindole [197, 200], is an ATP-competitive inhibitor of *Mtb* PS. It occupies the P2 site (used for the binding of pyrophosphate and β-alanine) of *Mtb* PS, and its OMe group and sulfone oxygen make hydrogen bonds with the backbone nitrogen of Val187, and both the backbone amide group of Met40 and the side chain of His47, respectively (Fig. 4i). (3) 2-[(1-benzofuran-2-ylsulfonyl) carbamoyl]-5-methoxy-1H-indol-1-yl-acetic acid (inhibitor 3) [197], is a product of fragment-linking of 5-methoxyindole and 1-benzofuran-2-carboxylic acid [201, 202]. The binding mode of indole acyl sulfonamide moiety of inhibitor 3 is similar to that of inhibitor 2. The benzofuran group is found at the P1 site (used for the binding of pantoate), and its carboxyl group makes hydrogen bonds with the Met40 and His47 of the enzyme (Fig. 4j) [197] and could also function as a pantoate-competitive inhibitor.

#### Enzymes involved in amino acid metabolism

##### Lysine-*ε* amino transferase (LAT)

*Mtb* has a remarkable capacity for persistence in the human host, causing latent infection in a quarter of the world’s population [203]. As an abnormally expressed gene during the stationary and non-replicating persistence phase of *Mtb*, LAT is upregulated by 41.86 times in in vitro models of tuberculosis [204–206]. A large number of research results have demonstrated the essential role of LAT in contributing to the long-term persistence of *Mtb*, and it may be listed as a fascinating potential target for latent tuberculosis [207]. Functionally, this enzyme is a pyridoxal-5′-phosphate (PLP)-dependent type II aminotransferase [208], which participates in the metabolism of L-lysine in a variety of organisms and catalyzes reversible transamination reactions from L-lysine to α-ketoglutaric acid, producing piperidine-6-carboxilic acid and L-glutamate [209–211].

**Fig. 5:**
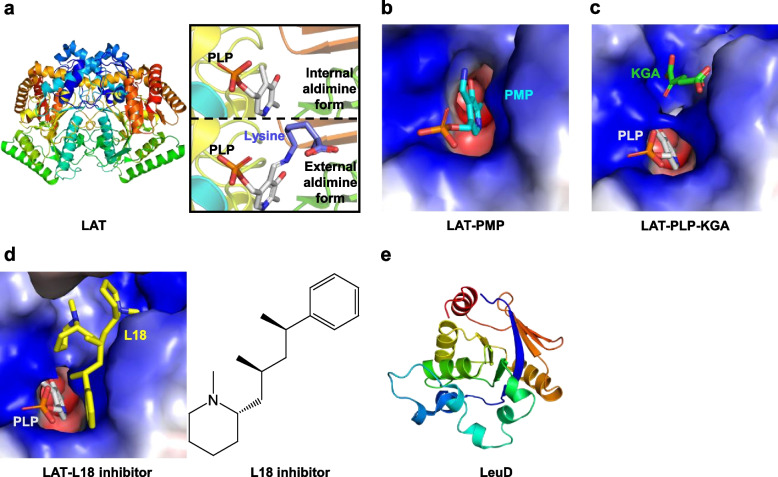
Enzymes associated with amino acid metabolism. **a** Structures of lysine-ε amino transferase (LAT) in the internal aldimine form (PDB ID: 2CIN) and external aldimine form (PDB ID: 2CJD). PLP, pyridoxal-5′-phosphate. **b**, **c** Electrostatic surface of LAT in complex with substrate PMP (PDB ID: 2CJG) and KGA in the external aldimine form (PDB ID: 2CJH), respectively. PMP, pyridoxamine 5′-phosphate; KGA, α-ketoglutarate. **d** Electrostatic surface of LAT in complex with inhibitor L18 (PDB ID: 2JJG). L18, (2S)-1-methyl-2-[(2S,4R)-2-methyl-4-phenylpentyl]piperidine. **e** N-terminal structure of isopropylmalate isomerase (LeuD1–186, PDB ID: 3H5H). The electrostatic potential in all figures was computed using the APBS tools in PyMol (http://www.pymol.org/)

The structure of apo-*Mtb* LAT is a homodimer, which is maintained by polar interactions and water-mediated interactions between the interface [212]. The overall fold of *Mtb* LAT is conserved across many other members of this enzyme family [209, 213], which consists of a large and small domain with the co-factor sandwiched between them (Fig. 5a). The active site of LAT is composed of residues from both subunits, including Glu243, Arg422, Gln274, Lys300, Arg170, Phe167, Thr330, and Asn328, among which the latter two come from the symmetry-related subunit. In the internal aldimine (PLP-bound) form of LAT, PLP is located in a pocket created by several residues from two monomers (Gly128, Ala129, Phe167, His168, Glu238, Asp271, Val273, Gln274, and Lys300 from one subunit; Ser329 and Thr330 from another subunit). Various contacts occur between PLP and the enzyme, including the Schiff base linkage with the active site Lys300, the hydrogen bonding between N1 of PLP and conserved Asp271, and hydrogen bonding between the phosphate moiety of PLP and Thr330, Gly128, Ala129, and several water molecules. To accommodate the lysine substrate, the PLP moiety rotates by about 14° around N1. A similar conformational change also occurred in the PMP (pyridoxamine 5′-phosphate)-bound LAT complex structure (Fig. 5b). In the structure of LAT in complex with PLP and lysine substrate (external aldimine form), the lysine occupies a pocket created by Val63, Lys300, Ser329, and Thr330 through bidentate hydrogen bonds with Arg170, and forms a stable internal N-C covalent bond with PLP. Then, the Schiff base linkage between PLP and Lys300 is broken and replaced by the Lys300-Thr330 interaction. Subgroup II aminotransferases adopt a characteristic “Glu243 switch” mechanism in substrate selection and reaction specificity. In the complex structure of LAT-lysine-PLP, Glu243 shields the positively charged Arg422 by making a salt bridge with this residue, and its C_γ_ and C_δ_ atoms also engage in van der Waals interactions with the C_δ_ and C_ε_ atoms of the substrate. All of these interactions prevent interactions between the carboxylate group of the substrates and Arg422 and prevent the undesired transamination at the α-amino group of the substrate, thus providing substrate specificity. Compared with the external form of LAT, a significant conformational change of Glu243 is observed in the complex structure of LAT bound with C5 substrates (L-Glutamate or α-ketoglutarate, KGA) (Fig. 5c). The Glu243-Arg422 interaction is disrupted and substituted by an open configuration, which is favorable for the binding of C5 substrates. Structurally, the α-carboxylate group and γ-carboxylate group of α-ketoglutarate interacts with Arg422 and Arg170, respectively. The interactions with the conserved Asn328 and several water molecules are also contributing to the stability of the C5 substrate. In these structures of LAT, variable numbers of water molecules are observed in the enzyme's active site, which has been proposed to play an essential role in the stability of complex structures.

The crystal structure of LAT bound to substrates has been explored for structure-based drug discovery. Several hit inhibitors of LAT have been reported, including a thiazole derivative [214], a 2-aminomethyl piperidine derivative [215, 216], and benzothiazole-based inhibitors [217]. The binary structure of LAT/2-aminomethyl piperidine derivative [(2S)-1-methyl-2-(2S,4R)-2-methyl-4-phenylpentyl-piperidine, L18] inhibitor demonstrates that this inhibitor imitates the binding of C5 substrates (Fig. 5d) [215]. Further, the docking results of remaining potential inhibitors of LAT also demonstrate that all these compounds occupy the active-site cavity and have approximately the same binding mode as the ligand [214, 217]. In addition, all these compounds exhibit effective activity against dormant tuberculosis, similar to some drug candidates, such as (8-Hydroxy quinoline) [218], capreomycin [219], proteasome inhibitor oxathiazole-2-one derivative, 5-nitrothiazole derivatives [220] and some alanine dehydrogenase inhibitors against non-replicating *Mtb* [221].

##### Isopropylmalate isomerase (LeuD)

Bacteria can biosynthesize all twenty proteinogenic amino acids, including the nine essential amino acids required for the growth of mammals [222]. Among these amino acids, the biosynthetic pathways of three branched-chain amino acids (BCAAs L-isoleucine, L-leucine, and L-valine) are more effective than other amino acids. Only eight conserved enzymes are sufficient for the biosynthetic pathway of all three BCAAs, including four branched-chain aminotransferases (IlvB/N, IlvC, IlvD, IlvE) that are conserved in the synthesis of all three BCAAs. Additional three enzymes that only participate in the synthesis of L-Leucine (LeuA, LeuC/D, and LeuB), and IlvA is only involved in L-isoleucine biosynthesis. The BCAAs are necessary for the growth and survival of *Mtb* [223, 224], and enzymes participating in the biosynthetic pathway of BCAAs have been proposed as potential drug targets [222]. The advantages of targeting these enzymes are obvious. Firstly, the absence of similar pathways in mammals may reduce the toxicity of related drugs. Secondly, the inhibition of BCAAs not only impacts the metabolism of essential amino acids, but also affects some other pathways that use BCAAs. Therefore, the inhibition of enzymes within the BCAA biosynthetic pathways are thought to be a “death by a thousand cuts” strategy against pathogenic organisms [222]. Conversely, the destruction of genes involved in these pathways may result in stunted growth and infection damage [225, 226], so these special gene-auxotroph strains may be used as vaccines to prevent future pathogenic infection. For example, the deletion of *leuD* of *Mtb* produces an attenuated strain, which could protect wild-type mice from virulent *Mtb* infection, and its degree of protection was approximately the same as that of *M. bovis* BCG [27, 227]. Besides, a double auxotroph strain (∆*panCD*∆*leuCD*) is even more protective than ∆*leuD* alone [222]. *Mtb* LeuC and LeuD form a heterodimer to exert enzymatic activity in catalysis of the stereospecific conversion from α-isopropylmalate to β-isopropylmalate, requiring an iron-sulfur cluster ([4Fe-4S]) in its catalytic center [228].

Up to now, the complex structure of *Mtb* LeuCD has not been determined. Only several C-terminal truncations of LeuD have been reported, including LeuD^1–156^, LeuD^1–168^, and Leu^1–186^ (Fig. 5e) [229]. *Mtb* LeuD shares a 15% sequence identity with the C-terminal domain of mitochondrial aconitase, and its overall fold is a twisted β/β/α three-layer sandwich. There are two flexible fragments in LeuD. One is the substrate recognition loop (residues 30–37), wherein the residue Arg32 may play a critical role in substrate recognition by forming hydrogen bonds with the γ-carboxylate of α-isopropylmalate. The other includes substrate interaction residues (Gly74-Ser75-Ser76-Arg77) around the GSSR sequence motif. In addition to LeuD, the structures and mechanisms of *Mtb* LeuA [230], LeuB [231], and IlvE [232, 233] have also been well characterized.

#### Enzymes involved in metal uptake

##### Iron-dependent regulator (IdeR)

Metals play vital roles in many important biological processes, especially serving as virtually indispensable cofactors, affecting the viability and growth of living organisms. Iron is one such essential cofactor. Higher organisms obtain iron in tight complexes through iron storage and transport proteins (e.g. transferrin, lactoferrin, and ferritin). To get enough iron from their environment, bacteria have evolved an iron-uptake system, which is based on a variety of low molecular weight iron chelators known as siderophores, such as mycobactin and exochelin of *Mtb* [234]. In Gram-negative bacteria and certain Gram-positive bacteria with low GC content, the regulation of iron uptake is usually carried out by the ferric uptake regulator *Fur*, while in other Gram-positive bacteria and archaea with high GG content, iron homeostasis is usually controlled by its functional homologue *IdeR* (iron-dependent regulator) [235–239]. When intracellular iron levels reach the threshold value, the iron-activated IdeR binds to the operator regions of target genes to inhibit the transcription of these genes by blocking the incoming RNA polymerase, preventing the iron concentration from increasing to reach toxic levels [238–240]. Under the condition of metal starvation, the metal-free IdeR is inactive, and all iron uptake genes are activated. In *Mtb,* approximately 40 genes involved in iron uptake and metabolism are regulated by IdeR [238, 241]. For instance, in response to high intracellular iron concentration, the activated *Mtb* IdeR binds to the operator of *mbtA-mbtJ* gene cluster involved in the biosynthesis pathway of mycobactin [238, 242], thereby inhibiting the transcription of *mbtA-J* genes, as well as the synthesis of mycobactin, and the uptake of iron. Conversely, the activated IdeR also functions as a transcriptional activator for the expression of some iron-storage genes, such as *bfrA* and *bfrB* [238, 243]. In addition to the IdeR, *Mtb* has another representative metalloregulator MntR (Rv2788) [238, 244], which functions as a manganese-dependent transcription repressor, and is related to manganese homeostasis. Compared with the less defined MntR, the biological and structural characteristics of IdeR has been described in detail, and it has been regarded as an attractive anti-tuberculosis drug target for decades [239, 243, 245].

**Fig. 6:**
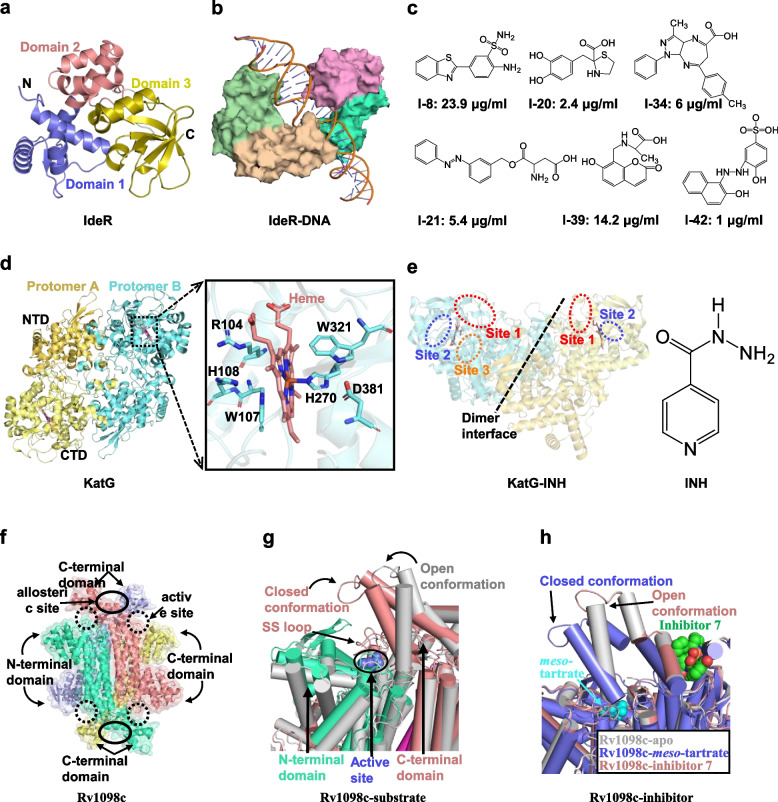
Enzymes associated with metal uptake and energy metabolism. **a** Structure of a subunit of IdeR homodimer (PDB ID: 1FX7). Domain 1, the N-terminal DNA-binding winged helix-turn-helix (wHTH) motif; domain 2, the dimerization domain; domain 3, the C-terminal SH3-like domain. **b** Structure of DNA- binding IdeR (PDB ID: 2ISZ). **c** Structure of small molecules potentially inhibiting IdeR activity and their respective IC50 values. **d** Structure of catalase-peroxidase (KatG) homodimer in a complex with heme (PDB ID: 2CCA). The heme is surrounded by six conserved residues, among which Arg104, Trp107, and His108 in a pocket distal to the heme, and His270, Trp321, and Asp381 in a pocket proximal to the heme. **e** Sites of INH binding to KatG (Left) and a chemical structure of isoniazid (INH, Right). The anti- tuberculosis pro-drug INH is required to be activated to form a bactericidal molecule (IN-NAD+ adduct) by KatG, and then targets the enoyl-acyl carrier protein reductase (InhA), which is responsible for biosynthesis of cell wall component mycolic acid. **f** Overall structure of the homotetrameric fumarate hydratase (Rv1098c, PDB ID: 4APA)). The dashed circles indicate the location of the four active sites, and the solid circles indicate the location of the two allosteric sites. **g** A depiction of Rv1098c active site when formate or L-malate (slate spheres) is bound. Apo-Rv1098c is shown in gray, while the N- and C-terminus of substrate (formate or L-malate)-bound Rv1098c are shown in greencyan and pink, respectively. Upon binding of formate or L-malate, the C-terminal domain of the subunit (purple) rotates into the closed conformation. **h** Superposition of apo (gray, PDB ID: 4APA), meso-tartrate-bound (slate, PDB ID: 4ADM) and inhibitor 7-bound (pink, PDB ID: 5F91) Rv1098c. Binding of meso-tartrate causes the active site of Rv1098c to be occupied, whereas binding of inhibitor 7 causes the C-terminus of the subunit to rotate into the open conformation

*Mtb* IdeR is a functional and structural homologue to the diphtheria toxin repressor (DtxR) from *Corynebacterium diphtheriae*, which can be substituted for each other in complementary experiments [246–249]. Apo-IdeR is very flexible, and it has a preferred monomer form over the dimer form [241, 250]. Extensive interactions occur between the two subunits of the IdeR homodimer. Each subunit consists of three domains, an N-terminal DNA-binding winged helix-turn-helix (wHTH) motif (residues 1–74; Domain 1), a dimerization domain (residues 75–140; Domain 2) consisting of three α-helices, and a C-terminal SH3-like domain (residues 151–230; Domain 3) consisting of six β-strands and three α-helices [251, 252] (Fig. 6a). A long helix (H4) connects Domain 2 and Domain 3. Compared with the other two domains, Domain 3 has low sequence conservation. It is structurally inserted into the groove between Domain 1 and 2 as a wedge and plays a significant role in stabilizing the active conformation of IdeR by providing ligands for metal-binding sites [251]. When activated cations are present, IdeR undergoes a complicated activation process, including metal-binding, dimerization, and coordination with specific promoter sequences of the targeted genes [250, 253, 254]. Distinguished from the apo structure of IdeR, conformational changes of two HTH motifs (especially two putative DNA-binding helices H3 and H3’; 6–9° rotation) are observed in the metal-activated IdeR. Overall, two DNA-binding helices get closer, and this conformational change is believed to be critical for these helices to be inserted into the major grooves of DNA [251, 255, 256]. In addition to the typical Fe^2+^ cofactor, several other divalent ions can also act as co-activators of IdeR in vitro, such as Co^2+^, Ni^2+^, Mn^2+^, Cd^2+^, and Zn^2+^ [246]. Several structures of IdeR bound with different metal ions (Co^2+^, Ni^2+^, Zn^2+^) have been determined. Except for some slight differences, these structures are nearly identical [241, 251, 255, 256]. In the crystal structure of metal-activated IdeR, there are two metal-binding sites, both are located at the interface between Domain 1 and Domain 2 (designated metal-binding site 1 and 2), with Domain 2 providing most of the ligands for the two metal-binding sites. Metal-binding site 1 is pentavalently coordinated by the side chains of residues His79, Glu83, and His98 from Domain 2, and side chains of Glu172 and Gln175 from Domain 3, and some non-protein ligands (a phosphate or sulfate ion, as well as a variable number of water molecules) [257, 258], forming a twisted octahedral geometry. Similar to metal-binding site 1, metal-binding site 2 is coordinated by six ligands, including the side chains of residues Met10, Clu105, His106, and Cys102, the main chain carbonyl oxygen of Cys102, and a water molecule that is linked to Leu4 of the N-terminal pentapeptide [253, 257]. Two metal-binding sites are bridged by hydrogen bonds (2.5 Å) formed by Glu105 and His79, so that each site can sense the effects of the other site. In some structures of activated-IdeR, the third metal (cobalt)-binding site located on the surface of Domain 3 was also observed, and this metal is coordinated by His219, His223, and four water molecules.

The conformational changes of IdeR resulting from metal ion binding primes IdeR to bind DNA. In the structures of nickel or cobalt-activated IdeR complex with *mbtA-mbtB* operator DNA, two homodimers of IdeR are bound to the opposite side of the DNA duplex, forming a “double-dimer” complex, in which the DNA duplex adopts a distorted *B*-DNA conformation with three complete helical turns, and metal-binding sites 1 and 2 completely occupied (Fig. 6b). Extensive contacts (hydrogen bonds, salt bridges, and van der Waals contacts) occurred between IdeR and the deoxyribose phosphate backbone and nucleotide bases of DNA. Most residues of IdeR involved in the interactions with DNA are mainly clustered in the HTH motif (residues 27–50), and this conserved motif inserts into the major groove of DNA, which is similar to other DtxR repressors. In addition, the wing of the HTH motif interacts with the DNA backbone on the minor groove edge, thus clamping the backbone between the wing and the first helix of the HTT motif. In the IdeR-DNA complex structure, a “*p*_*1*_*s*_*2*_*C*_*3*_*T*_*4*_*a*_*5*_” (*p*_*1*:_ purine; *s*_*2*_: cytosine or guanine; *C*_*3*:_ cytosine; *T*_*4*:_ thymine; *a*_*5*:_ adenine) base recognition pattern is regarded as the basis of key interactions between each IdeR protomer and DNA [251]. Two essential residues (Ser37 and Pro39) move by 1–3 Å to protrude into the major groove of DNA, and specifically interact with the T4 base through van der Waals contacts [251]. Residue Pro39 also interacts with nucleotide bases (at consensus positions + 3 and + 8) via additional van der Waals interactions. In addition, Gln43 forms many van der Waals contacts with various nucleotide bases on fingerprint positions *p*_*1*_ and *s*_*2*_ of the DNA [251]. Briefly, IdeR recognizes and utilizes the thymine base on position T4 as anchor points for base-specific recognition, and Gln43 of IdeR makes non-specific interactions with nucleotide bases on fingerprint positions *p*_*1*_ and *s*_*2*_ [251]. This structural information is of great value for structure-based drug discovery.

To date, there is no reported structure of IdeR in complex with inhibitors. Only several inhibitors [(I-8 (NSC65748), I-20 (NSC281033), I-21 (NSC30600), I-34 (NSC662444), I-39 (NSC673342), and I-42 (NSC12453)] of IdeR have been identified by initial virtual screening and later sets of biochemical validation (Fig. 6c) [245]. All 6 compounds show inhibitory activity on the DNA binding function of IdeR, among which I-20 and I-42 exhibit the most efficient inhibition ability (IC_50_ value is 2.4 μg/ml and 1 μg/ml, respectively). In the initial molecular docking, the structural information of IdeR was utilized, and the above-mentioned essential residues (Ser37, Pro39, and Gln43) and their adjacent residue (Ser42) was designated as the grid center (docking site) for docking the filtered NSC database. The predicted key interactions between IdeR and I-20 include hydrogen bonds formed between the benzyl ring or the carboxylic acid of I-20 and the carboxyl group of Gln43 and the amino group of Ser37, respectively [245]. It is predicted that Compound I-42 forms a hydrogen bond and a hydrogen bond network with the hydroxyl group of Ser42 and two amino groups of Arg60, respectively [245]. Previous studies on DtxR showed that almost all mutations that destroy the DNA-binding properties are located on the metal binding site or helix H3. Therefore, potential inhibitors targeting these regions can be screened.

#### Enzymes involved in energy metabolism

##### Catalase-Peroxidase (KatG)

During pathogenic infection, catalase-peroxidases protect aerobic microorganisms from oxidative damage. As the only catalase in *Mtb*, the heme-dependent catalase-peroxidase KatG degrades hydrogen peroxide (H_2_O_2_) and organic peroxides to escape the attack of reactive oxygen intermediates from the host [259, 260]. Aside from catalase and non-specific peroxidase activity, KatG concurrently possesses manganese peroxidase, oxidase, INH-hydrazinolysis, and isonicotinoyl (IN)-NAD synthase activities [261, 262]. INH, an anti-tuberculosis pro-drug, needs to be activated by KatG to form a bactericidal molecule (IN-NAD^+^ adduct) and then target InhA. It has been reported that over 60% of known INH-resistant mutations are associated with *katG* [263–266], and other non-lethal mutations are found within *inhA* [267]. Therefore, understanding the relationship between INH-mediating mutations in KatG and their effects on the structure and mechanism of INH activation is critical to settle the growing incidence of INH-resistant TB infection.

The structure of KatG has been determined by X-ray crystallography [268] or cryo-EM [269], and its structure is similar to peroxidases in many bacteria and plants (Fig. 6d) [270–272]. The homo-dimeric structure is predominantly α-helical, with two domains in each protomer. The N-terminal domain is homologous to the C-terminal domain, while only the former domain contains b-type heme, which is essential for enzyme function. This heme is pentacoordinated and surrounded by six conserved residues, among which Arg104, Trp107, and His108 are in a pocket distal to the heme, and His270, Trp321, and Asp381 in a pocket proximal to the heme. The homodimer is linked by a hook-like structure composed of the N-terminal residues of both protomers [268]. Two important structural elements for enzymatic activity are listed below. The first one is a covalently linked MYW catalytic triad, which consists of three conserved residues, Met255, Tyr229, and Trp107, and is required for catalase activity [273–275]. The second is a substrate entry channel, of which the bottleneck is delimited by residues Asp137 and Ser315 (the diameter of the bottleneck is 3.6 Å), resulting in a steric restriction for access to the heme active site. Although many studies have shown that the activation of INH depends on high-valent (ferryl) heme in KatG, the confirmed binding site of INH within KatG has not been identified to date. This may be related to the transient and dynamic interaction of INH and KatG, which leads to a widespread distribution of binding site. Recently, a cryo-EM structure of KatG bound to INH revealed several potential binding sites of INH (designated as site 1, site 2, site 3) (Fig. 6e). Structurally, the addition of INH did not perturb KatG’s heme site, and the heme environment of KatG-INH complex was the same as that of apo-KatG. Site 1 exists in both subunits and is situated at the entrance to the distal heme pocket (δ-edge of the heme). This binding site is close to residues Ser315 and Asp137, both of which were reported to regulate the activation of INH [276, 277]. Site 2 also exists in both subunits, and it is situated near the γ-edge of the heme. The third INH binding site is only observed in protomer B and is situated toward the dimer-dimer interface, and two amino acids implicated with INH resistance (Gly299 and Trp300) are adjacent to this binding site [265, 266, 278].

Finally, in order to explore how KatG mutation affects the activation of INH, several typical INH-resistance mutant residues near the edge of heme (such as Ser315Thr [279, 280], Asp137Ser [276], Trp107Arg, Thr275Pro [281]) are described. Firstly, most prevalent INH-resistant *Mtb* strains carry KatG^S315T^, and their INH affinity to KatG^S315T^ is lower than that of WT KatG (about 40-fold) [282]. Compared with the structure of WT KatG, the significant conformational change of KatG^S315T^ results in the size of the bottleneck in the substrate channel to become reduced from 3.6 Å to 2.7 Å [276, 283], which is induced by the methyl group of Thr315. Contrarily, the KatG^D137S^ mutant exhibits greatly improved INH-activation catalysis ability compared to that of WT KatG (Km value; 192 μM vs 17.5 μM) [284]. In the structure of the KatG^D137S^ mutant, an expanded entry channel was observed (4.6 Å). Therefore, the change in size of the bottleneck in the substrate channel of KatG may increase or decrease the INH peroxidation activity. Secondly, as a catalytic residue, the replacement of Trp107 causes the loss of catalase activity [266], while it still retains the peroxidase activity. Within the cryo-EM structure of KatG^W107R^, each homodimer of protein has only one bound heme. There is no heme in the protomer A, and there is obvious structural disorder near the heme binding site. This heme deficiency caused by the Trp107Arg mutation could be supplemented by exogenous heme supplements (aminolevulinic acid and hemin chloride) [269]. Likewise, another INH-resistance mutation (Thr275Pro) also leads to a lower heme occupancy. This structural information provides an in-depth insight into INH resistance.

##### Fumarate hydratase (Rv1098c)

According to respective structural characteristics, the ubiquitous fumarate hydratase (fumarase), which catalyzes the reversible conversion from fumarate to L-malate during the TCA, may be classified into two subgroups: class I (homo-dimeric) and class ΙΙ (homo-tetrameric) [285]. Unlike other bacteria, *Mtb* has only one fumarase (Rv1098c), making it a vulnerable and attractive therapeutic target for drug development against *Mtb* [8, 223, 286]. However, the high sequence identity (53%) and the same active site shared between human fumarase and *Mtb* fumarase pose a challenge in developing anti-tuberculosis drugs targeting this enzyme [287].

The overall structure of Rv1098c displays a symmetric homo-tetramer conformation, which shares significant structural similarity with other members of the class II fumarase superfamily [288] (Fig. 6f). Each dumbbell-shaped subunit contains three domains: an N-terminal domain (residues 1 to 137), a large central α-helix domain (residues 138 to 393), and a small C-terminal domain (residues 394 to 466). A central, elongated 20-helix bundle is created by five tightly packed helices at the center of each subunit, and is capped by two small N-terminal and C-terminal domains, which are also predominantly composed of α-helices. Four symmetry-related active sites of fumarase are positioned at a cleft, which is formed by residues from three subunits and covered by a “SS loop” (residues Pro316-Val325). It has been demonstrated that this loose loop plays a crucial role in ligand binding and enzymatic activity [289, 290], especially the catalytic residue Ser318 [288]. Compared with the apo structure of Rv1098c, both the complex structure of Rv1098c/L-malate or Rv1098c/fumarate undergo a remarkable conformational change, including the swing of SS-loop and a rigid-body movement (inward bending by about 34°) of the C-terminal domain, which leads to the closure of the active sites (Fig. 6g). Structurally, only two ligands were observed in the four available enzyme active sites. One of the substrate molecules forms a series of hydrogen bonding interactions with Ser104, Thr106, Ser138, Ser139, and Asn140 of subunit A, with Thr186 and His187 of subunit B, and Ser318, Ser319, Lys324, and Asn326 of subunit C, while the other one forms equivalent hydrogen bonds with the corresponding residues of subunits B, A, D [288].

Several inhibitors of Rv1098c have been identified [291], including competitive and allosteric inhibitors. A well-known competitive inhibitor of fumarases is *meso*-tartrate. In the crystal structure of Rv1098c with *meso*-tartrate (Fig. 6h), the *meso*-tartrate molecules are bound at two enzyme active sites in a manner similar to the binding of substrates. On the other hand, the first allosteric inhibitor of Rv1098c, designated as inhibitor 7 ([N-(5-(azepan-1-ylsulfonyl)-2-methoxy-phenyl)-2-(4-oxo-3,4-dihydrophthalazin-1-yl) acetamide]), was identified by combination with high-throughput screening and structure validation [292]. This inhibitor shows a high selective inhibitory ability of *Mtb* fumarase, but has no effect on the human homolog [292]. In addition, inhibitor 7 could inhibit the growth rate in *Mtb* H37Rv strain in a dose-dependent manner. In the structure of Rv1098c in complex with inhibitor 7, two identical non-conservative allosteric sites (site 1 and site 2) were observed at the interface of two C-terminal domains of subunits A (C) and B (D). However, only allosteric site 1 was fully occupied, and site 2 may be related to crystal contact. The π-π stacking (between the two core phenyl rings of two inhibitors) and several other contacts, such as hydrogen bond and stacking interactions (between the inhibitors and surrounding residues), collectively anchor these two inhibitor molecules in this allosteric site, which is 7 to 20 Å away from each of the two nearest active sites. The binding of inhibitor 7 results in a dramatic conformational change, where the C-terminal domain rotates outward to align with the substrate-free enzyme (in the open conformation). This conformational change induced by the allosteric inhibitor is distinguished from that of substrate- or competitive inhibitor-binding, where the C-terminal domain rotates inward for 34° compared with the unbound form of Rv1098c. Therefore, these selective inhibitors drive significant conformational changes of Rv1098c, and then competitively prevent the substrate and inhibitor from binding at the neighboring active sites by locking the subunits in the open conformation. Other allosteric inhibitors (inhibitor 1 and its derivates) have also been reported [291]. Similarly, these inhibitors dimerically bind to the allosteric site of Rv1098c, and lock the nearest active site in an open conformation. These lead compounds could be optimized by studying structure-activity relationships. On the other hand, the hit compounds should have bactericidal activity against *Mtb*.

#### Enzymes involved in nucleotide biosynthesis

##### Cytidine triphosphate synthetase (PyrG)

The high-energy compound cytidine triphosphate (CTP) is involved in various metabolic processes and impacts cell growth as well as ATP [293]. PyrG, ATP-dependent CTP synthetase, is responsible for catalyzing the amination of uridine triphosphate (UTP) to form CTP in the last step of the pyrimidine nucleotide biosynthesis pathway [294]. In *Mtb,* pharmacological inhibition of PyrG could interfere with DNA/RNA biosynthesis, and other nucleotide-dependent metabolic processes, such as the biosynthesis of fatty acids, carbohydrates, amino acids, and cAMP or c-di-AMP [295]. PyrG, the essential gene within *Mtb,* has been regarded as a potential drug target [295, 296].

**Fig. 7:**
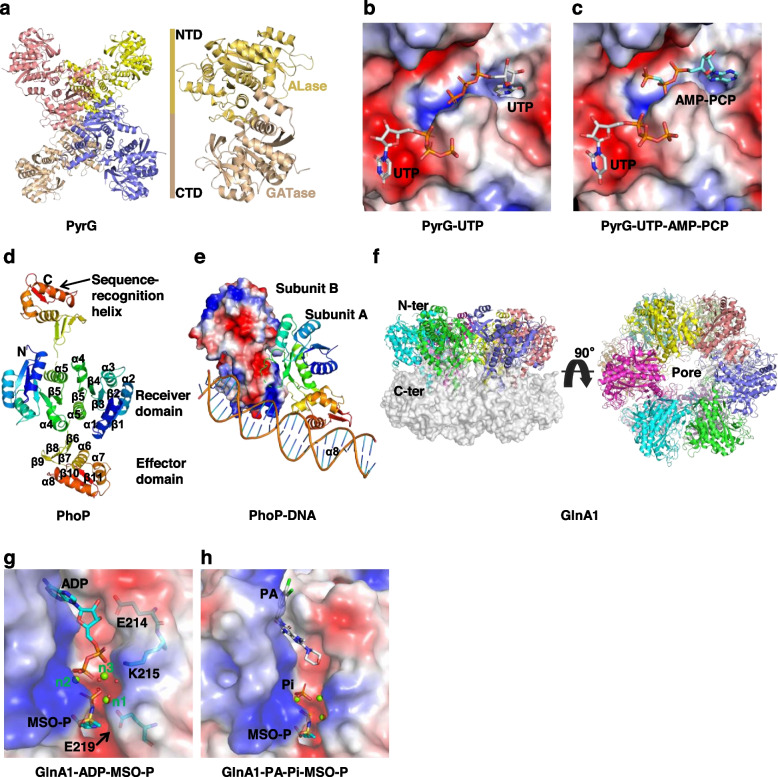
Proteins associated with nucleotide biosynthesis and transcriptional regulation. **a** Tetrameric structure of cytidine triphosphate synthetase PyrG-apo (PDB ID: 4ZDI). The N-terminal synthetase (ALase) domain is positioned at the center of the tetramer while the C-terminal glutaminase (GATase) domain is pointing outwards. **b**, **c** Electrostatic surface of PyrG in a complex with nucleotides and analogs, i.e. either UTP or UTP/AMP-PCP. **d** Structure of PhoP. PhoP (PDB ID: 3R0J) dimerizes through α4-β5-α5 of the receiver domain with a 2-fold symmetry. The N- and C-termini for both subunits are marked with N and C, respectively. **e** Structure of PhoP-DNA complex. Ribbon diagram of the PhoP- DNA complex (PDB ID: 5ED4) shows a tandem PhoP dimer binding to a directly repeating DNA sequence. The two PhoP subunits are designated A and B, with subunit A binding to the first TCACAGC motif that is directly repeated, while B binds to the second motif. The subunits A and B are represented by cartoon and electrostatic surfaces, respectively. **f** Oligomeric structure of GlnA1 (PDB ID: 1HTQ) is a double symmetric structure formed by hexamers in an asymmetric unit. Each subunit - subunit interface has 12 active sites. **g** Electrostatic surface of GlnA1 in a complex with MSO-P and ADP (PDB ID: 2BVC). **h** Electrostatic surface of GlnA1 in complex with MSO-P, PA and Pi (PDB ID: 2WHI). The electrostatic potential in all figures was computed using the APBS tools in PyMol (http://www.pymol.org/)

The structure of apo-PyrG consists of an N-terminal amidoligase (ALase) domain (referred to as the synthetase domain; residues 1 to 278), and a C-terminal glutamine amido-transferase (GATase) domain (residues 299 to 552) (Fig. 7a) [295]. The two domains are composed of nearly identical Rossmann-like folds, which are connected by an interdomain linker (residues 279–298). However, the presence of bound molecules (UTP, or UTP/ATP analog AMP-PCP/glutamine analog 5-oxo-L-norleucine) change the oligomeric state of PyrG from monomer to tetramer. The ATP- and UTP-binding pockets located on the concave surface of PyrG are defined by residues from two and three adjacent subunits, respectively (Fig. 7b-c). The active site of PyrG glutaminase is indicated by the characteristic GATase catalytic triad (Cys393-His524-Glu526) [294, 295, 297]. In addition, a putative ammonia diffusion channel, which is located between the active site of glutaminase and the amidoligase domain, provides an entrance point for exogenous ammonia.

Two thiophenecarboxamide derivatives, compound 7,947,882 [5-methyl-N-(4-nitrophenyl)-thiophene-2-carboxamide] and 7,904,688 [3-phenyl-*N*-(4-piperidin-1-ylphenyl)-carbamothioyl-propanamide], which require activation by the monooxygenase EthA, may kill *Mtb* by inhibiting PyrG [295]. The EthA-activated metabolite of compound 7,947,882 (compound 11,426,026) could directly inhibit the activity of PyrG. Docking the compound 11,426,026 into PyrG indicates that it only recognizes the ATP-binging site of PyrG, in which the phenyl ring forms π-π stacking with Arg223, and its nitro group forms hydrogen bonds with Ala253 and Asp252 [295]. In addition, a series of 4-(pyridine-2-yl)-thiazole derivatives also have the ability to inhibit PyrG [298]. All these compounds appear to function as competitive inhibitors of the ATP binding site. Interestingly, some recent studies showed that two prodrugs 7,947,882 and 7,904,688, and the compound 11,426,026 have a second target, the pantothenate kinase PanK, which participates in the biosynthesis of coenzyme A [299, 300]. Therefore, this suggests that these direct PyrG and PanK inhibitors should be used as lead compounds of multi-target antitubercular drugs, and these two proteins are potentially to be as a “double-tool” for hit compound screening [299].

### Transcriptional regulators

#### Response factor PhoP

In bacteria, groups of two-component signal transduction systems (TCSs) mediate various signal processes (e.g. sporulation, transformation competence, membrane transport, stress response, and virulence), which are absent in mammals [301, 302]. Most TCSs consist of a sensor histidine kinase and a response regulator (RR), wherein histidine kinase senses environmental signals and auto-phosphorylates on a conserved histidine residue, transferring the phosphate group to a conserved aspartate residue of cognate RR, thus regulating gene transcription to generate cellular response [232]. *Mtb* encodes 30 TCSs, including 11 systems and 7 histidine kinases or RRs [8, 233], among which the PhoP-PhoR system has the greatest impact on *Mtb* virulence [303–305]. The absence of the *phoP* or the *phoR* severely weakens the virulence of *Mtb* strains [306–309], and these attenuated strains are being developed into live vaccines [310, 311]. In the PhoP-PhoR system, PhoR functions as a transmembrane histidine kinase to transmit environmental signals, and PhoP regulates transcription by binding to protomer DNA of corresponding genes [312, 313]. *Mtb* PhoP may regulate the expression of more than 110 genes [307], especially those related to lipid biosynthesis [307, 314]. Therefore, exploring the mechanism of the PhoP-PhoR system and the structural information of its components will contribute to the development of antituberculosis drugs.

*Mtb* PhoP is a member of the OmpR/PhoB subfamily, which is the largest subfamily of RRs [232, 315]. This protein contains two distinct domains, an N-terminal receiver domain (residues 1–138) and a C-terminal DNA-binding domain (also known as effector domain; residues 150–247) (Fig. 7d). The receiver domain consists of a central five-stranded parallel β-sheets (β1-β5), sandwiched by helices on both sides [316]. An acidic pocket composed of several acidic residues (Asp27, Asp28, Glu29, and Asp71) was presented at the C-terminal ends of strands β1 and β3. In this pocket, the residue Asp71 is responsible for phosphorylation [313]; and it is hydrogen bonded by the conserved residue Lys121, which may contribute to dephosphorylation/phosphorylation reactions [317]. There is still confusion about how phosphorylation of the receiver domain regulates the DNA-binding activity of PhoP through its effector domain. One prevalent view considers that the phosphorylation of the receiver domain promotes or stabilizes PhoP dimerization, thus bringing the effector domain near the DNA direct repeat [318–322]. The phosphorylated PhoP forms a dimer through the α4-β5-α5 face of the receiver domain, and is stabilized by multiple interactions (including π-electron stacking, charge-charge interactions, salt bridges, and hydrogen bonds). Conversely, the conformational changes of switch residues Thr99 and Tyr118 were considered to be the response of phosphorylation. Upon phosphorylation, the side chain of Thr99 is oriented away from the acidic pocket, and Tyr118 is in an inward conformation facing toward the phosphorylation site. Contrarily, Tyr118 is in outward conformation in the unphosphorylated active dimers [317]. The side chain of Tyr118 also participates in the interactions of the dimer interface [317].

Compared with the receiver domain, the effector domain shows a great degree of flexibility. The isolated effector domain exists primarily as a monomer in solution, but forms a hexamer ring in crystal via tandem association between adjacent protomers, and two hexamers are linked by the crystallographic 2-fold symmetry to generate a dodecamer [323]. The effector domain consists of three α-helices flanked by two β-sheets, including a four-stranded antiparallel β-sheet (β6-β9) at the N-terminus, and a three-stranded antiparallel β-sheet formed by the C-terminal β-hairpin (β11-β12) and a short strand between helices α6 and α7. This effector domain has a typical winged helix-turn-helix fold of the OmpR/PhoB subfamily of RRs [323], in which the helix-turn-helix motif is formed by helices α7 and α8, and the wing motif is formed by the C-terminal β-hairpin turn. A long and flexible loop connects the receiver and effector domain together. This loop is necessary for phosphorylation-dependent DNA binding [312], and may play a role in phosphorylation signaling between two domains [313, 317, 324].

Distinguished from the structure of apo-PhoP, a symmetric receiver domain dimer connects to a tandem effector domain dimer [317], a new conformation of the PhoP-DNA complex has been found [325]. In the structure of the PhoP complex with DNA (Fig. 7e), a DNA duplex was bound to a highly synergistic tandem dimer (both receiver and effector domains were in tandem association). Two effector domains interact with DNA in the same way, and their contact areas with DNA are nearly identical. The effector domain binds to DNA by recognizing direct repeats of 7 bp motifs with a 4 bp spacer. Structurally, the outward-facing side chains of residues (Asn212, Val213, Glu215, Ser216, Tyr217, and Tyr220) in the sequence-recognition helix α8 interact with the base of TCACAGC motif in the major groove of DNA through hydrogen bonds, π-π stacking, hydrophobic, and van der Waals interactions [323]; and the residues (Arg237, Gly238, Thr235) of wing structure interact with the adjacent minor groove. This binding pattern of DNA is consistent with the electrostatic potential on the protein surface. The electrostatic potential of the recognition helix and the wing residues is extremely positive, while most of the remaining parts are negatively charged or neutral, which cause the protein to orient to initially bind to the DNA duplex. Collectively, this available structural information provides preliminary insight for the development of inhibitors against PhoP.

### Other potential targets

To date, approximately 200 secreted proteins are detected in the *Mtb* culture medium [326]. Several filtrate proteins (GlnA1, Esat6/CF-10 [327], LpqH, HspX) [141, 328] found in the early stages of infection are also promising targets for anti-tuberculosis drugs. Besides, some other potential targets are also listed in this manuscript (Table 2).

**Table 2:** Overview of potential targets of anti-tuberculosis agents

No.	Genes (Rv numbers)	Protein name	Description
1	*pks13* (Rv3800c) [329]	Polyketide synthase Pks13	Synthesis of mycolic acid
2	Rv1885c	Chorismate mutase *(Mtb*CM) [330]	Synthesis of shikimate (rearrange chorismate to prephenate)
3	*alr* (Rv3423c)	Alanine racemase [331]	Synthesis of cell wall (racemase L-alanine into D-alanine)
4	*glfT1* (Rv3782) *glfT2* (Rv3808c)	Galactofuranosyl transferase, GlfT [332]	Biosynthesis of galactan
5	*rmID* (Rv3266c)	dTDP-6-deoxy-L-*lyxo*-4-hexulose reductase, RmID [333]	Biosynthesis of L-rhamnosyl
6	*eccB3* (Rv0283)	EccB3 [334]	A component of the ESX-3 type VII secretion system
7	*aspS* (Rv2572c) [329]	Aspartyl-tRNA synthetase, AspS	Protein translation
8	*fadD32* (Rv3801c) [335]	Fatty acyl-AMP ligase, FadD32	Synthesis of mycolic acid (links the FAS and PKS mycolate pathways)
9	*dfrA* (Rv2763c)	Dihydrofolate reductase DHFR [336]	Synthesis of nucleic acid
10	*accD4* (Rv3799c)	AccD4-containing acyl-CoA carboxylase [337]	Biosynthesis of mycolic acids
11	*fabH* (Rv0533c) [338]	3-oxoacyl-ACP synthase III, FabH	Synthesis of mycolic acid (responsible for initiation of FAS II fatty acid biosynthesis)
12	*mabA/fabG1* (Rv1483)	β-ketoacyl-ACP reductase, MabA [339]	A complex group of enzymes responsible for the production of very long fatty acid derivatives
13	*coaA* (Rv1092c) [340]	Pantothenate kinase (PanK)	Biosynthesis of CoA (catalyzes the first and rate-limiting step of the CoA biosynthesis)
14	*aroK* (Rv2539c)	Shikimate kinase [341]	Biosynthesis of chorismate
15	*ahpD* (Rv2429) [342]	Alkylhydroperoxidases AhpC and AhpD	Catalyzes the reduction of alkylhydroperoxides to alcohols
16	*ribH* (Rv1416) [343]	Lumazine synthase (LS)	Biosynthesis of riboflavin (catalyzes the formation of 6,7-dimethyl-8-D-ribityl-lumazine)
17	*nrdR* (Rv2718c)	Ribonucleotide reductases [344],	Catalyzing the formation of deoxyribonucleotides from ribonucleotides
18	*ligA-D* (Rv3014c, Rv3062, Rv3731, Rv0938)	DNA ligase [345]	Replication of DNA
19	*tmk* (Rv3247c)	Thymidine monophosphate kinase (TMPKmt) [346]	Phosphorylate thymidine monophosphate to thymidine diphosphate
20	*atpA-atpH* (Rv1308, Rv1304, Rv1311, Rv1310, Rv1305, Rv1306, Rv1309, Rv1307)	ATP synthase [347]	Production of ATP
21	*ndh* (Rv1854c)	NADH-menaquinone oxidoreductase [348]	Biosynthesis of menaquinone
22	*alas, argG, argS, aspS, cysS1, gltS, glyS, hisS, ileS, leuS, lysS, lysX, metS, pheS, pheT, proS, serS, thrS, trpS, tyrS, valS, acs, menE, birA, mshC, panC, guaA, nadE, mbtA, mbtB, mbtE, mbtF, nrp, fadD1–19, fadD21–26, fadD27–36* [349]	Adenylate-forming enzymes (AEs) [349]	Activation of carboxylic acids to intermediate acyladenylates
23	*dprE1* (Rv3790) [350]	Flavoenzyme DprE1	Cell wall synthesis (catalyzes the epimerization of decaprenyl-phospho-ribose to decaprenyl-phospho-arabinose)
24	*gyrA* (Rv0006), *gyrB* (Rv0005)	DNA gyrase [351]	DNA replication (regulates DNA topology)
25	*clpP1*(Rv2461); *clpP2* (Rv2460c )[352]	Clp protease (ClpP1 and ClpP2)	Degradation of misfolded or damaged proteins
26	*prsA* (Rv1017c) [353]	Phosphoribosyl pyrophosphate synthetase	Biosynthesis of phosphoribosyl-1-pyrophosphate
27	*hspX or acr* (Rv2031c)	HspX [354]	An alpha-crystallin-like protein, which associates with the growth suppression of *Mtb*
28	*hupB* (Rv2986c)	Nucleoid-associated protein HU [355]	Contributes to the maintenance of chromosomal structure and the global regulation of DNA transactions

#### Glutamine synthetase (GlnA1)

Glutamine synthetase GlnA1 (also called γ-glutamyl: ammonia ligase) catalyzes the condensation of ammonium and glutamate to generate glutamine, whose activity depends on ATP and divalent cations (magnesium or manganese ions) [356]. Multiple metabolites of GlnA1 (glutamine, glutamate, and poly-L-glutamate-glutamine) play important roles in the nitrogen metabolism, and osmoregulation; and also serve as the essential constituent of the cell wall of mycobacteria [357, 358]. Therefore, GlnA1 is a promising anti-tuberculosis target.

The apo structure of GlnA1 is a dodecamer stacked by two hexamers face to face [356] (Fig. 7f), and the active sites of GlnA1 are formed by two adjacent subunits (referred to as “bifunnel”). After binding of metal ions and ATP, GlnA1 converts from a relaxed (inactive) to a taut (active) state [356, 359]. Compared to the relaxed state, the most striking difference is a three-residue register shift of the β-strand consisting of Glu214, Lys215, and Glu219 [359]. Glutamate analogues, L-methionine-*SR*-sulfoximine (MSO), and phosphinothricin have been shown to selectively inhibit GlnA1 and disrupt the development of bacterial cell walls and consequently inhibit the growth of *Mtb* [357], but do not affect nonpathogenic mycobacteria or nonbacterial microorganisms [357]. In the structure of the GlnA1 complex with phosphorylated-MSO (MSO-P), Mg, and ADP [359], ADP and MSO-P are located on both sides of the “bifunnel”, and three metal ions (n1, n2, n3) participate to stabilize the complex (Fig. 7g). Some purine analogues (e.g. 1-[(3,4-dichlorophenyl) methyl]-3,7-dimethyl-8-morpholin-4-yl-purine-2,6-dione) termed as PA, and 2-tert-butyl-4,5-diarylimidazoles which act as ATP-competitive inhibitors of GlnA1 have been identified [358, 360]. In both the crystal structure of GlnA1/PA, and GlnA1/PA/MSO-P/Mg (Fig. 7h), which represent the active and inactive conformation of GlnA1, respectively, PA occupies the position of ADP-ribose [358]. The binding mode of PA with *Mtb* GlnA1 is different from that of the human GlnA1, in which the dichlorophenyl group of the PA will clash with the side chains of Trp130 and Arg262 of human GlnA1 [358]. Likely, PA can be reasonably used as a lead compound to design potent and selective inhibitors. In addition, using special antisense oligonucleotides to interfere with the activity of GlnA1 has also been proposed to treat tuberculosis [361].

## Conclusion and prospect

Although TB chemotherapy and the BCG vaccine are readily available, tuberculosis still causes considerable morbidity and mortality annually across the globe, highlighting an urgent need for new medicine against *Mtb*, especially resistant and/or persistent strains. New therapeutic strategies may arise from a better understanding of the molecular basis of the metabolic pathways. Thus, it necessitates the identification of essential genes or virulence factors of *Mtb*, which are significant for the survival and growth of the bacilli. Furthermore, mechanistic insights into the multiplication and intracellular persistence of *Mtb* within the infected host are also required.

Here, this review summarized several validated and promising drug targets, exploring their structure and structure-based drug/inhibitor designs. Those enzymes are involved in multiple cellular metabolic pathways, including fatty acid biosynthesis, and some other pathways (the metabolism of lipids, amino acids, energy utilization, metal uptake, nucleotide biosynthesis, and transcriptional regulation). All these essential enzymes are closely related to the pathogenesis and drug resistance of mycobacteria. Anti-tuberculosis drugs targeting these essential genes would provide an opportunity for us to develop novel, structurally diverse and promising compounds to eradicate the TB disease. In this work, we also listed two enzymes that are related to the persistent phase of *Mtb*, Isocitrate lyases (ICL1 and ICL2) and Lysine-ε aminotransferase (LAT). These two enzymes are abnormally expressed during the persistence phase of *Mtb*, and drugs targeting these targets are expected to solve the problem of persistent TB infection.

## Data Availability

Not applicable.
